# Shotgun proteomics deciphered age/division of labor-related functional specification of three honeybee (*Apis mellifera* L.) exocrine glands

**DOI:** 10.1371/journal.pone.0191344

**Published:** 2018-02-15

**Authors:** Toshiyuki Fujita, Hiroko Kozuka-Hata, Yutaro Hori, Jun Takeuchi, Takeo Kubo, Masaaki Oyama

**Affiliations:** 1 Department of Biological Sciences, Graduate School of Science, The University of Tokyo, Bunkyo-ku, Tokyo, Japan; 2 Medical Proteomics Laboratory, The Institute of Medical Science, The University of Tokyo, Minato-ku, Tokyo, Japan; 3 Institute of Molecular and Cellular Biosciences, The University of Tokyo, Bunkyo-ku, Tokyo, Japan; 4 Medical Research Institute, Tokyo Medical and Dental University, Bunkyo-ku, Tokyo, Japan; Leibniz Institute on aging - Fritz Lipmann Institute (FLI), GERMANY

## Abstract

The honeybee (*Apis mellifera* L.) uses various chemical signals produced by the worker exocrine glands to maintain the functioning of its colony. The roles of worker postcerebral glands (PcGs), thoracic glands (TGs), and mandibular glands (MGs) and the functional changes they undergo according to the division of labor from nursing to foraging are not as well studied. To comprehensively characterize the molecular roles of these glands in workers and their changes according to the division of labor of workers, we analyzed the proteomes of PcGs, TGs, and MGs from nurse bees and foragers using shotgun proteomics technology. We identified approximately 2000 proteins from each of the nurse bee or forager glands and highlighted the features of these glands at the molecular level by semiquantitative enrichment analyses of frequently detected, gland-selective, and labor-selective proteins. First, we found the high potential to produce lipids in PcGs and MGs, suggesting their relation to pheromone production. Second, we also found the proton pumps abundant in TGs and propose some transporters possibly related to the saliva production. Finally, our data unveiled candidate enzymes involved in labor-dependent acid production in MGs.

## Introduction

Animals live in a world of environmental stimuli that affect their life histories; *i*.*e*., their developmental trajectories, as well as their physiologic and/or behavioral states. Polyphenism is typical of such biologic phenomena where animals that have the same genomes exhibit distinct morphologic, physiologic, and/or behavioral phenotypes depending on the environmental stimuli that the animals perceive during developmental stages [1]. In some cases, such environmental stimuli are produced by the animals *per se*. The caste differentiation of Hymenopteran insects, especially honeybees, is one of the most well-characterized examples of polyphenism, where the food produced by nestmates affects the fate of other nestmates [2, 3].

The European honeybee (*Apis mellifera* L.) is a social insect that lives in a colony containing approximately 30,000–100,000 individuals, and whose sophisticated society is sometimes likened to a ‘superorganism’. Only one queen is engaged in laying eggs as a reproductive caste, while the other female bees, workers, are engaged in various labors to maintain colony activity as a labor caste [4]. In addition, workers shift their labors according to their age after eclosion: young workers (nurse bees: approximately 6–12 days after emergence) are mainly engaged in nursing the brood and queen by secreting royal jelly (RJ), whereas older workers (foragers: older than 14 days after emergence) are mainly engaged in collecting nectar and pollen outside the hive [2].

Honeybee females differentiate into queens or workers from the same eggs depending on the food fed to them after birth by the nurse bees. The key component of the food for queen determination is RJ, food rich in protein and lipid that is synthesized and secreted by nurse bees. Larvae fed a rich RJ diet differentiate into queens, whereas larvae fed a poor RJ and rich honey diet differentiate into workers [2, 3]. The chief proteinous component of RJ comprises the major royal jelly proteins (MRJPs). Although Kamakura reported that Royalactin, also known as MRJP1 [5], is responsible for queen determination, the RJ factor(s) important for queen determination remains controversial [6–8].

In nurse bees, the hypopharyngeal glands (HpGs), which open in the mouthpart, are considered to synthesize and secrete the proteinous components of RJ, whereas mandibular glands (MGs), which open in the mandible, synthesize and secrete the lipid components [2]. We previously demonstrated that not only the HpGs, but also the salivary glands, comprising the head postcerebral glands (PcGs) and thoracic glands (TGs), which connect to a common duct and open in the mouthpart [9, 10], contribute to produce RJ proteinous components [11, 12]. Among these worker exocrine glands, HpGs have been extensively studied, because they are expected to synthesize the factor(s) responsible for queen determination and their roles change according to the division of labor of the workers: while nurse bee HpGs predominantly synthesize MRJPs, forager HpGs synthesize and secrete carbohydrate-metabolizing enzymes that process nectar into honey [13–20]. Therefore, the physiologic states of the worker HpGs reflect the labors of the workers. In contrast, however, knowledge regarding the functions of the PcGs, TGs, and MGs remains limited.

We previously performed a gel-based proteomic analysis of the worker salivary glands (PcGs and TGs) to identify the characteristic protein expression patterns of these glands [11]. Expression of both aldolase and acetyl CoA-acyltransferase 2, which are involved in glycolysis and fatty acid metabolism, respectively, is enhanced in the PcGs compared with the TGs, especially in foragers [11, 12], consistent with the previous report that the honeybee primer pheromone ethyl oleate (EO) was found to be present in the PcGs [21], suggesting that EO is synthesized in the PcGs [11, 12].

The previous studies by our group and others regarding the labor-related functions of honeybee exocrine glands, however, were based on small-scale gel-based proteomic analyses [11, 22]. In addition, the preceding shotgun proteomic studies of PcG and TG functions did not analyze labor-related functional specification of the glands: *i*.*e*., mixtures of nurse bee and forager PcGs and TGs were used as samples [12, 23]. In the present study, to expand our understanding of the labor-dependent functional specifications of these exocrine glands, we performed large-scale shotgun proteomic measurements and detailed pathway analyses of PcGs, TGs, and MGs dissected from nurse bees and foragers.

## Results

### Proteomic identification of the three exocrine glands of worker honeybees

**Fig 1:**
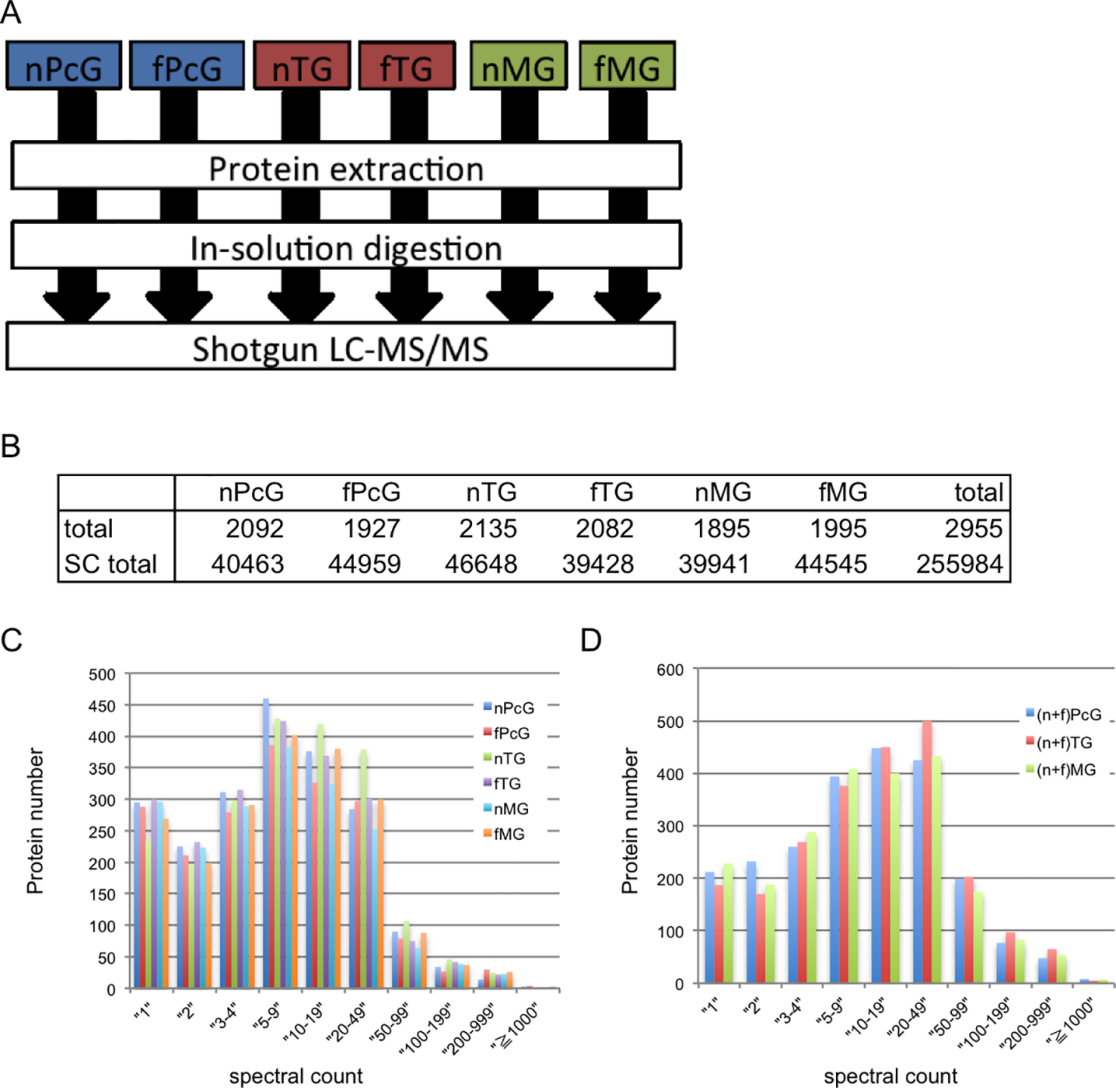
Experimental overview of the shotgun proteomics strategy. (A) The protein extracts from the three exocrine glands separated according to division of labor; nPcGs, fPcGs, nTGs, fTGs, nMGs, and fMGs, were in-solution digested and subjected to shotgun LC-MS/MS [67]. (B) The number of proteins identified according to the criteria described in the *Methods* section and the total number of spectral counts detected in each gland (nPcG, fPcG, nTG, fTG, nMG, and fMG) are shown. SC, spectral count. (C) Distribution of spectral counts for the six glands: nPcG, fPcG, nTG, fTG, nMG, and fMG. (D) Distribution of spectral counts for each gland based on the data merged from nurse bees and foragers in panel (C).

In this study, to characterize the division of labor-related functions of the three exocrine glands of worker honeybees, we extracted proteins from the PcGs of nurse bees (nPcGs) and foragers (fPcGs), the TGs of nurse bees (nTGs) and foragers (fTGs) as well as the MGs of nurse bees (nMGs) and foragers (fMGs), digested them with trypsin, and subjected them to a direct nanoflow LC-MS/MS system, as illustrated in Fig 1 (Fig 1A).

As a result, 2092, 1927, 2135, 2082, 1895, and 1995 proteins were identified from the nPcGs, fPcGs, nTGs, fTGs, nMGs, and fMGs, respectively, leading to detection of 2955 proteins in total (Fig 1B, S1–S7 Tables). The numbers of the proteins identified and the spectral counts detected from each gland were approximately 2000 and 40,000, respectively (Fig 1B). The distributions of the spectral counts were also similar to each other among the six glands (Fig 1C) and the trend was the same when the spectral counts from the nurse and forager glands were merged (Fig 1D), suggesting that our large-scale mass spectrometric measurement worked stably at the proteomic scale. Therefore, we considered that the spectral counts obtained in our study were applicable for the subsequent semiquantitative analyses. We then applied these 2955 identified proteins to the KEGG pathway analyses [24], which we previously used for our proteomic analysis of the functions of HpGs, PcGs, and TGs [12], for gland-selective and labor-related functional characterization.

### Functional characterization of the three exocrine glands based on the frequently detected proteins

The numbers of the proteins identified from these exocrine glands in the present study were at least 2-fold higher than those obtained in previous studies [11, 12, 22, 23], possibly due to the high resolution of our MS instrument. Therefore, to further deepen our understanding of the functions of these exocrine glands, we first analyzed their proteomes at the gland level by merging the spectral counts regarding the nurse bee and forager bee glands (Fig 1D).

**Fig 2:**
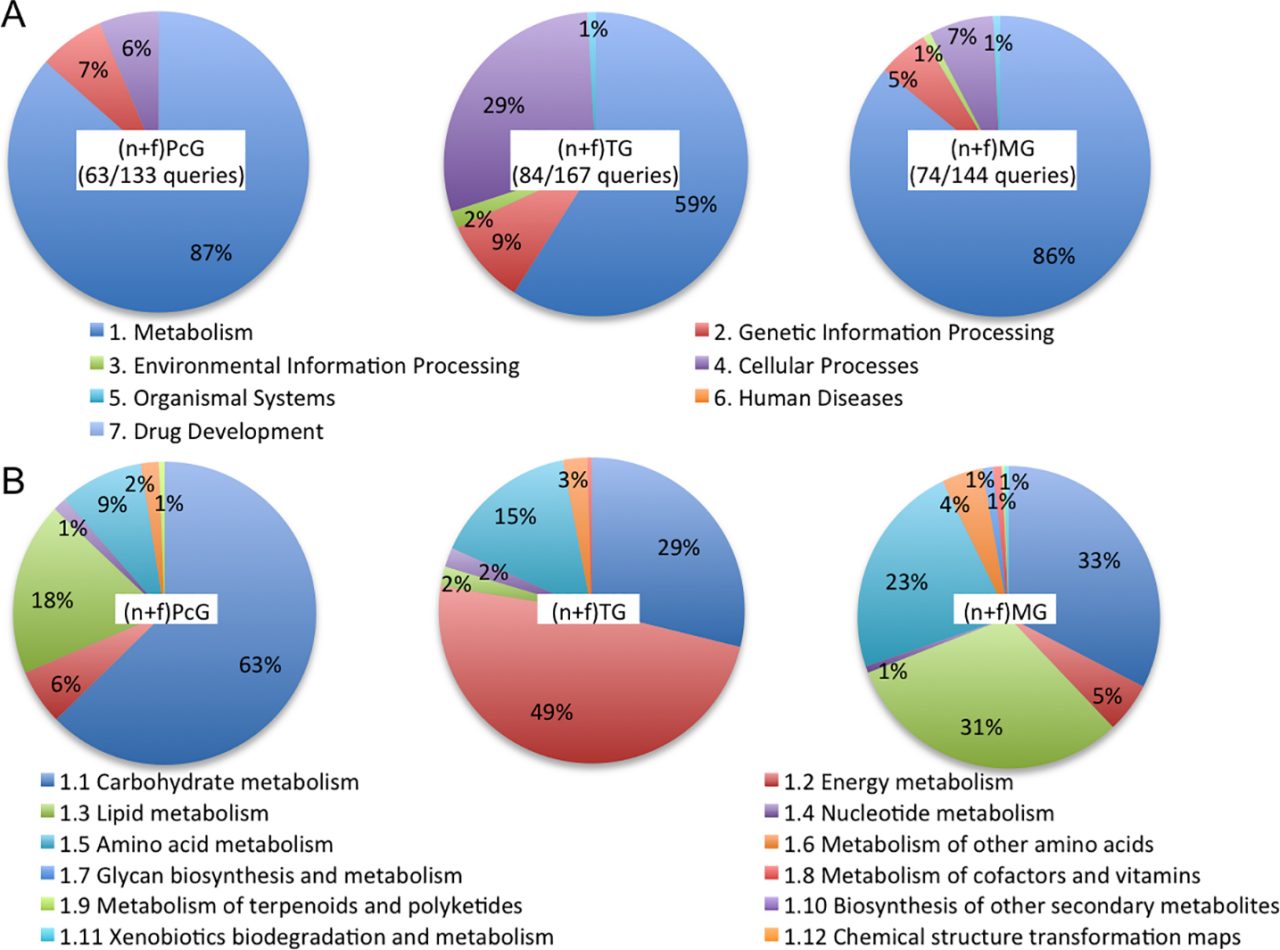
Functional analysis of the three exocrine glands based on the frequently detected proteins identified from the PcGs, TGs, and MGs. (A) Semiquantitative comparison of the KEGG pathway categories of the frequently detected proteins identified from the PcGs, TGs, and MGs. (B) Semiquantitative comparison of the KEGG pathway subcategories of the frequently detected proteins categorized as “Metabolism” in panel (A).

When we pooled the proteins identified from each of the nurse bee and forager glands, 133, 167, and 144 proteins were identified from (n+f)PGs, (n+f)TGs, and (n+f)MGs, whose spectral counts were more than 100 (partially illustrated in Table 1 and the full lists are provided in S8 Table). All but 1 of the top 10 frequently detected proteins from the PcGs and TGs in our previous shotgun proteomics analysis [12] were included in the list of the top 30 frequently detected proteins, indicating that our previous results are reproducible even in this scaled-up study; the one exception was fibrillin-2-like in the TGs, which was the 8^th^ most frequently detected protein in our previous study, but not included among the top 30 frequently detected proteins in the present study (Table 1 and S8 Table). Among these 133, 167, and 144 proteins, the KEGG pathway analysis enabled us to map 63, 84, and 74 proteins to some functional categories and each enrichment p-value was provided as S9 Table and S1 Fig. When their spectral counts were assigned to each category, the largest category corresponded to “Metabolism” (87%, 59%, and 86% for (n+f)PcGs, (n+f)TGs, and (n+f)MGs, respectively) (Fig 2A and S9 Table). When we further categorized the “Metabolism” category into subcategories, the largest category differed among the three glands (Fig 2B). The largest and second largest categories of (n+f)PcGs corresponded to “Carbohydrate metabolism” (63%) and “Lipid metabolism” (18%), those of (n+f)TGs corresponded to “Energy metabolism” (49%) and “Carbohydrate metabolism” (29%), and those of (n+f)MGs corresponded to “Carbohydrate metabolism” (33%) and “Lipid metabolism” (31%) (Fig 2B). Notably, “Lipid metabolism” comprised only 2% of (n+f)TGs, suggesting that lipid metabolism was not the major function of (n+f)TGs. In addition, in accordance with the fact that “Carbohydrate metabolism” was the largest subcategory in both of the (n+f)PcGs and (n+f)MGs and the second largest in the (n+f)TGs, the numbers of the frequently detected proteins in “Carbohydrate metabolism” for PcGs, TGs, and MGs were 71, 44, and 74, respectively (S10 Table), suggesting that carbohydrate metabolism was more active in the PcGs and MGs than in the TGs.

**Table 1:** Top 30 frequently detected proteins identified from each gland.

		**Spectral count**			
**Accession**	**Description**	**nPcG**	**fPcG**	**(n+f)PcG**	**Fujita, *et al*. 2013**
gi|328789361	p. transketolase isoform 1	2207	2084	4291	3
gi|66503776	p. transketolase isoform 2	2208	2082	4290	6
gi|328787941	p. fatty acid synthase-like	1016	2821	3837	1
gi|66530142	p. ATP-citrate synthase isoform 1	583	1387	1970	2
gi|48142692	p. glyceraldehyde-3-phosphate dehydrogenase 2 isoform 1	491	785	1276	16
gi|110748949	p. fructose-bisphosphate aldolase-like	368	887	1255	10
gi|66547450	p. 60 kDa heat shock protein, mitochondrial-like	662	499	1161	9
gi|328785411	p. acetyl-CoA carboxylase-like	370	645	1015	5
gi|48097100	p. 3-ketoacyl-CoA thiolase, mitochondrial-like	461	488	949	15
gi|328785025	p. ATP synthase subunit beta, mitochondrial	367	511	878	8
gi|328788708	p. pyruvate carboxylase, mitochondrial-like	217	451	668	4
gi|297591985	actin related protein 1	359	267	626	31
gi|297591987	actin related protein 1	359	267	626	31
gi|300807169	fatty acyl-CoA reductase 1	151	380	531	14
gi|328778238	p. NADP-dependent malic enzyme isoform 1	153	351	504	7
gi|336391183	ATP synthase lipid-binding protein, mitochondrial	291	210	501	-
gi|336391185	ATP synthase lipid-binding protein, mitochondrial	291	210	501	-
gi|328777397	p. alkyldihydroxyacetonephosphate synthase-like	239	231	470	34
gi|328786450	p. ATP synthase subunit delta, mitochondrial isoform 1	185	242	427	143
gi|328786452	p. ATP synthase subunit delta, mitochondrial isoform 2	185	242	427	143
gi|66517317	p. ATP synthase subunit delta, mitochondrial isoform 3	185	242	427	143
gi|48100966	p. ATP synthase subunit alpha, mitochondrial isoform 1	165	255	420	11
gi|66531797	p. ubiquitin-40S ribosomal protein S27a	150	266	416	307
gi|328783160	p. transaldolase	141	261	402	23
gi|110756311	p. ubiquitin-60S ribosomal protein L40 isoform 1	141	259	400	182
gi|328780726	p. polyubiquitin-A-like	141	259	400	182
gi|328780728	p. polyubiquitin-A-like	141	259	400	182
gi|328786389	p. pyruvate kinase-like	183	217	400	12
gi|328790046	p. ubiquitin-60S ribosomal protein L40 isoform 2	141	259	400	182
gi|328790050	p. ubiquitin-60S ribosomal protein L40 isoform 4	141	259	400	182
		**Spectral count**			
**Accession**	**Description**	**nTG**	**fTG**	**(n+f)TG**	**Fujita, *et al*. 2013**
gi|66531434	p. v-type proton ATPase subunit B-like	1329	1517	2846	4
gi|66515272	p. v-type proton ATPase catalytic subunit A-like isoform 1	1267	1132	2399	3
gi|58531215	ADP/ATP translocase	732	845	1577	6
gi|48094573	p. hypothetical protein LOC408608	803	719	1522	9
gi|328785025	p. ATP synthase subunit beta, mitochondrial	636	631	1267	7
gi|66556287	p. v-type proton ATPase subunit E isoform 3	441	442	883	33
gi|297591985	actin related protein 1	394	400	794	20
gi|297591987	actin related protein 1	394	400	794	20
gi|66553147	p. v-type proton ATPase subunit G	401	286	687	100
gi|328777212	p. spectrin alpha chain-like	360	323	683	1
gi|62526112	elongation factor 1-alpha	339	268	607	20
gi|328787929	p. spectrin beta chain	302	274	576	5
gi|48100966	p. ATP synthase subunit alpha, mitochondrial isoform 1	287	267	554	10
gi|328792073	p. v-type proton ATPase subunit H isoform 2	296	248	544	13
gi|328783869	p. basement membrane-specific heparan sulfate proteoglycan core protein-like	363	175	538	2
gi|328787562	p. tubulin alpha-1 chain-like	260	251	511	14
gi|66535209	p. tubulin alpha-1 chain-like	261	243	504	78
gi|328786450	p. ATP synthase subunit delta, mitochondrial isoform 1	257	241	498	107
gi|328786452	p. ATP synthase subunit delta, mitochondrial isoform 2	257	241	498	107
gi|66517317	p. ATP synthase subunit delta, mitochondrial isoform 3	257	241	498	107
gi|66515294	p. v-type proton ATPase subunit D 1-like isoform 1	269	210	479	148
gi|66547450	p. 60 kDa heat shock protein, mitochondrial-like	252	222	474	11
gi|336391183	ATP synthase lipid-binding protein, mitochondrial	213	230	443	589
gi|336391185	ATP synthase lipid-binding protein, mitochondrial	213	230	443	589
gi|48142692	p. glyceraldehyde-3-phosphate dehydrogenase 2 isoform 1	215	211	426	29
gi|66531797	p. ubiquitin-40S ribosomal protein S27a	193	188	381	240
gi|110756311	p. ubiquitin-60S ribosomal protein L40 isoform 1	182	184	366	240
gi|328780726	p. polyubiquitin-A-like	182	184	366	240
gi|328780728	p. polyubiquitin-A-like	182	184	366	240
gi|328790046	p. ubiquitin-60S ribosomal protein L40 isoform 2	182	184	366	240
		**Spectral count**			
**Accession**	**Description**	**nMG**	**fMG**	**(n+f)MG**	
gi|328787941	p. fatty acid synthase-like	2118	4263	6381	
gi|48097100	p. 3-ketoacyl-CoA thiolase, mitochondrial-like	2682	1140	3822	
gi|201023353	esterase A2	881	1128	2009	
gi|328789361	p. transketolase isoform 1	748	644	1392	
gi|66503776	p. transketolase isoform 2	745	640	1385	
gi|297591985	actin related protein 1	374	705	1079	
gi|297591987	actin related protein 1	374	705	1079	
gi|332801003	acyl-CoA synthetase family member 2, mitochondrial precursor	682	121	803	
gi|328785025	p. ATP synthase subunit beta, mitochondrial	348	373	721	
gi|66547450	p. 60 kDa heat shock protein, mitochondrial-like	399	208	607	
gi|295849268	superoxide dismutase 1	411	194	605	
gi|66548355	p. acyl-CoA synthetase family member 2, mitochondrial-like, partial	535	0	535	
gi|328786450	p. ATP synthase subunit delta, mitochondrial isoform 1	248	253	501	
gi|328786452	p. ATP synthase subunit delta, mitochondrial isoform 2	248	253	501	
gi|66517317	p. ATP synthase subunit delta, mitochondrial isoform 3	248	253	501	
gi|66523006	p. probable cytochrome P450 6a14 isoform 1	239	245	484	
gi|66511554	p. glucosylceramidase-like isoform 1	111	369	480	
gi|328785411	p. acetyl-CoA carboxylase-like	229	225	454	
gi|328785413	p. acetyl-CoA carboxylase-like isoform 2	229	225	454	
gi|110760701	p. 3-hydroxyacyl-CoA dehydrogenase type-2-like	315	131	446	
gi|336391183	ATP synthase lipid-binding protein, mitochondrial	269	176	445	
gi|336391185	ATP synthase lipid-binding protein, mitochondrial	269	176	445	
gi|66530142	p. ATP-citrate synthase isoform 1	259	160	419	
gi|328783869	p. basement membrane-specific heparan sulfate proteoglycan core protein-like	120	296	416	
gi|48100966	p. ATP synthase subunit alpha, mitochondrial isoform 1	194	218	412	
gi|328783535	p. transitional endoplasmic reticulum ATPase TER94 isoform 1	171	235	406	
gi|399220320	cuticular protein 28 precursor	66	329	395	
gi|328785290	p. probable cytochrome P450 6a14	208	186	394	
gi|110757387	p. ecdysteroid UDP-glucosyltransferase-like	133	258	391	
gi|110756311	p. ubiquitin-60S ribosomal protein L40 isoform 1	176	208	384	

The partial list of frequently detected (Spectral counts>100) proteins identified from the nPcGs, fPcGs, nTGs, fTGs, nMGs, and fMGs. The numbers in ‘Fujita, *et al*. 2013’ column indicate semiquantitative ranking of frequently detected proteins in our previous shotgun proteomics analysis of each gland. The full lists of the identified proteins and frequently detected proteins from the (n+f)PcGs, (n+f)TGs, and (n+f)MGs are provided as S7 and S8 Tables, respectively. The data are partially cited from Fujita *et al*. (2013) [12] with permission. Copyright (2013) American Chemical Society. p., predicted. Note that we did not normalize the spectral count of each protein with its predicted molecular mass, and thus used the words ‘frequently detected proteins’ in the text for the proteins listed in this Table.

While the frequently detected proteins subcategorized in both “Carbohydrate metabolism” and “Lipid metabolism” for PcGs and MGs were quite similar, the list of the frequently detected proteins for the TGs was remarkably different. Interestingly, two enzymes involved in fatty acid biosynthesis: fatty acid synthase-like and acetyl-CoA carboxylase-like; three enzymes involved in fatty acid elongation: 3-ketoacyl-CoA thiolase, mitochondrial-like, which is consistent with our previous report [11] (in our previous report this enzyme was called acetyl CoA-acyltransferase 2), trifunctional enzyme subunit beta, mitochondrial-like, and trifunctional enzyme subunit alpha, mitochondrial-like; and two enzyme involved in fatty acid degradation: probable medium-chain specific acyl-CoA dehydrogenase, mitochondrial-like, and trifunctional enzyme subunit beta, mitochondrial-like, were frequently detected in both of the PcGs and MGs, but not in the TGs (Table 2), suggesting that lipid metabolism is more active in the PcGs and MGs than in the TGs. In addition, alkyldihydroxyacetonephosphate (alkyl DHAP) synthase-like was exclusively detected in the PcGs and glucosylceramidase-like isoform 1 was almost exclusively detected (more than 100-fold higher) in the MGs (Table 2), suggesting that ether lipid metabolism and sphingolipid metabolism pathways are selectively upregulated in the PcGs and MGs, respectively.

**Table 2:** List of the frequently detected proteins categorized in ‘Lipid metabolism’ from the three exocrine glands.

			Spectral count		
Pathway	Accession	Description	(n+f)PcG	(n+f)TG	(n+f)MG
1.3 Lipid metabolism					
Fatty acid biosynthesis / ame00061					
	gi|328787941	p. fatty acid synthase-like	3837	51	6381
	gi|328785411	p. acetyl-CoA carboxylase-like	1015	45	454
Fatty acid elongation / ame00062					
	gi|48097100	p. 3-ketoacyl-CoA thiolase, mitochondrial-like	949	70	3822
	gi|66507594	p. trifunctional enzyme subunit beta, mitochondrial-like	114	82	113
	gi|328778689	p. probable enoyl-CoA hydratase, mitochondrial	77	39	107
	gi|66519936	p. trifunctional enzyme subunit alpha, mitochondrial-like	173	131	168
Fatty acid degradation / ame00071					
	gi|66499429	p. probable medium-chain specific acyl-CoA dehydrogenase, mitochondrial-like	141	64	380
	gi|328778689	p. probable enoyl-CoA hydratase, mitochondrial	77	39	107
	gi|66519936	p. trifunctional enzyme subunit alpha, mitochondrial-like	173	131	168
	gi|66530423	p. aldehyde dehydrogenase, mitochondrial isoform 1	84	75	349
	gi|66507594	p. trifunctional enzyme subunit beta, mitochondrial-like	114	82	113
Synthesis and degradation of ketone bodies / ame00072					
	gi|66535270	p. succinyl-CoA:3-ketoacid-coenzyme A transferase 1, mitochondrial-like	97	72	109
	gi|48141273	p. hydroxymethylglutaryl-CoA synthase 1	132	57	34
Glycerolipid metabolism / ame00561					
	gi|66530423	p. aldehyde dehydrogenase, mitochondrial isoform 1	84	75	349
Glycerophospholipid metabolism / ame00564					
	gi|62526114	glycerol-3-phosphate dehydrogenase	88	101	92
	gi|328784660	p. glycerol-3-phosphate dehydrogenase, mitochondrial-like	79	104	111
Ether lipid metabolism / ame00565					
	gi|328777397	p. alkyldihydroxyacetonephosphate synthase-like	470	0	0
Sphingolipid metabolism / ame00600					
	gi|66511554	p. glucosylceramidase-like isoform 1	4	0	480
Biosynthesis of unsaturated fatty acids / ame01040					
	gi|66519936	p. trifunctional enzyme subunit alpha, mitochondrial-like	173	131	168

‘Pathway’ represents KEGG pathway category. Spectral counts indicate the peptide numbers of the proteins identified in each gland. The shaded numbers in ‘Spectral count’ column satisfy our frequently detected protein criteria. p., predicted. Note that we did not normalize the spectral count of each protein with its predicted molecular mass, and thus used the words ‘frequently detected proteins’ in the text for the proteins listed in this Table.

In contrast to the PcGs and MGs, “Energy metabolism” was the largest subcategory of “Metabolism” in the TGs (Fig 2B), consistent with our previous finding [12]. We found, in the present study, that 11 subunits of vacuolar (V-) ATPases among 17, 6 of which were included in the TG top 30 list, were frequently detected proteins in the “Energy metabolism” category of the TGs (Table 1 and S8 Table). V-ATPase is a large multi-subunit complex ATP-dependent proton pump composed of an ATP-hydrolytic domain (V_1_) and a proton-translocation domain (V_0_) [25], suggesting that ATP-dependent membrane transport is active in the TGs.

### Functional characterizations of the three exocrine glands based on the ‘gland-selective’ proteins

**Fig 3:**
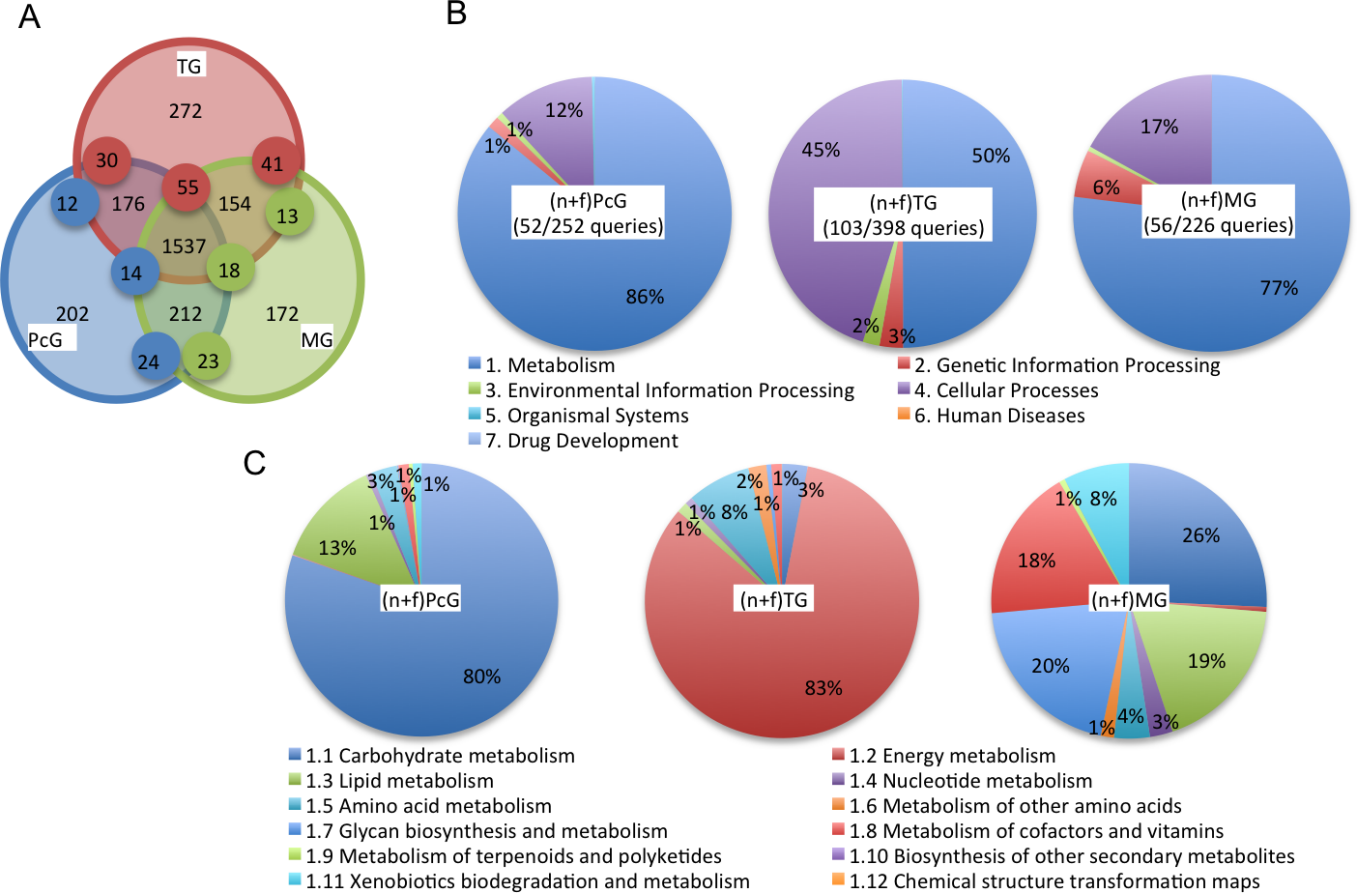
Functional analysis of the three glands based on the gland-selective proteins identified from the PcGs, TGs, and MGs. (A) The venn diagram shows the numbers of the proteins specifically detected from the glands and the small circles expanded on the boundaries indicate an abundance at least 5-fold higher than the same-color circles. The number of the corresponding proteins is indicated in each partition. (B) Semiquantitative comparison of the KEGG pathway categories of the gland-selective proteins identified from the PcGs, TGs, and MGs. (C) Semiquantitative comparison of the KEGG pathway subcategories of the gland-selective proteins categorized as “Metabolism” in panel (B).

While frequently detected proteins reflect major functions of the glands, ‘gland-selective’ proteins are thought to relate to the ‘gland-selective’ functions. Therefore, we next focused on ‘gland-selective’ proteins of these three exocrine glands by subtracting proteins overlapping with each other from those identified from each of the three exocrine glands: (n+f)PcGs, (n+f)TGs, and (n+f)MGs. We identified 202, 272, and 172 proteins that were specifically detected in (n+f)PcGs, (n+f)TGs, and (n+f)MGs, respectively (spectral counts >2). In addition, by normalizing the spectral count by the total spectral count in each gland, we selected proteins whose relative spectral counts in each gland were far greater than 5-fold more than that in the other glands as ‘gland-selective’ proteins. In total, 252, 398, and 226 proteins were identified as ‘PcG-, TG-, and MG-selective’ proteins, respectively (Fig 3A and S11 Table).

We then performed a pathway analysis using the KEGG Mapper tool with NCBI-gi as queries, and mapped 52, 103, and 56 proteins among 252 PcG-selective, 398 TG-selective, and 226 MG-selective proteins, respectively (S11 Table). Enrichment p-values were provided as S12 Table and S2 Fig. As a result, the largest category corresponded to “Metabolism” in all three glands; each corresponded to 86%, 50%, and 77% of the total spectral counts for the PcGs, TGs, and MGs, respectively (Fig 3B). We then further evaluated the “Metabolism” subcategories.

In the PcGs, the largest subcategory corresponded to “Carbohydrate metabolism” (80%), which was larger than that in the TGs (3%) and MGs (26%) (Fig 3C). The second largest subcategory of PcGs corresponded to “Lipid metabolism” (13%). Especially, alkyl-DHAP synthase-like was specifically and frequently detected (top 18 frequently detected) in the PcGs, suggesting that the “Ether lipid metabolism” pathway is upregulated in the PcGs (Table 2 and S12 Table). In contrast, the “Cellular processes” category (45%) was larger in TGs than in the other two glands (12% and 17%, respectively), reflecting the fact that V-ATPases are most frequently detected in TG and redundantly categorized in both “Metabolism” and “Cellular processes” (S12 Table). In the MGs, the top three categories corresponded to “Carbohydrate metabolism” (26%), “Glycan biosynthesis and metabolism” (20%), and “Lipid metabolism” (19%) (Fig 3C). Especially, glucosylceramidase-like isoform1 accounted for large portions of both the “Glycan biosynthesis and metabolism” (89.6%) and “Lipid metabolism” (97.6%) categories (S12 Table), suggesting their important roles in MG function. The overall signature of the PcG- and MG-selective KEGG pathway analysis was similar, suggesting their similar functions in lipid-metabolism.

### V-ATPases as selectively and frequently detected proteins in the TGs

Among the three exocrine glands, TGs were unique in that their largest subcategory of the “Metabolism” corresponded to “Energy metabolism”, while those in PcGs and MGs corresponded to “Carbohydrate metabolism” (Fig 3B). Therefore, we next focused on the “Energy metabolism” subcategory of TG, in which V-ATPase proteins were most prominent (S12 Table). First, we manually picked all V-ATPase proteins from the total list of proteins identified in the TGs. Among them, 12 V-ATPase proteins: v-type proton ATPase catalytic subunit A-like isoform 1, v-type proton ATPase subunit B-like, v-type proton ATPase subunit C, v-type proton ATPase subunit D 1-like isoform 1, v-type proton ATPase subunit E isoform 3, v-type proton ATPase subunit F 1-like, v-type proton ATPase subunit G, v-type proton ATPase subunit H isoform 1, v-type proton ATPase 116kDa subunit a isoform 1-like isoform 1, v-type proton ATPase subunit d, v-type proton ATPase subunit e 2-like, and v-type proton ATPase 21 kDa proteolipid subunit-like, fulfilled our criteria for ‘TG-selective proteins’ and were mapped using a KEGG mapper tool (S12 Table).

Among the unmapped V-ATPase proteins, three V-ATPase proteins: v-type proton ATPase subunit H isoform 2, v-type proton ATPase 116 kDa subunit a isoform 1-like, and vacuolar H+ ATP synthase 16 kDa proteolipid subunit also fulfilled our criteria for ‘TG-selective proteins’ and the other two V-ATPase components: v-type proton ATPase subunit C-like and probable V-type proton ATPase 116 kDa subunit a-like were more frequently detected in TGs than in the other two glands (Table 3). As a whole, nearly every V-ATPase protein was TG-selective, although the F_1_F_0_ ATP synthases, which are structurally and mechanistically related to the V-ATPases [26], were not differentially expressed among the PcG, TG, and MG samples, except ATP synthase subunit s, mitochondrial-like (S13 Table), suggesting important functions of V-ATPases in the TGs.

**Table 3:** List of the identified proteins relative to v-type proton ATPase from the three exocrine glands.

			Spectral count					
Accession		Description	nPcG	fPcG	nTG	fTG	nMG	fMG
*V1 domain*								
	gi|66515272	p. v-type proton ATPase catalytic subunit A-like isoform 1	52	68	1267	1132	77	139
	gi|66531434	p. v-type proton ATPase subunit B-like	61	67	1329	1517	74	120
	gi|328781744	p. v-type proton ATPase subunit C	7	6	116	113	12	6
	gi|328781786	p. v-type proton ATPase subunit C-like	6	6	28	21	4	10
	gi|66515294	p. v-type proton ATPase subunit D 1-like isoform 1	4	6	269	210	16	22
	gi|66556287	p. v-type proton ATPase subunit E isoform 3	32	32	441	442	32	34
	gi|66529931	p. v-type proton ATPase subunit F 1-like	0	6	210	117	2	4
	gi|66553147	p. v-type proton ATPase subunit G	16	12	401	286	33	34
	gi|328792071	p. v-type proton ATPase subunit H isoform 1	0	0	284	0	41	0
	gi|328792073	p. v-type proton ATPase subunit H isoform 2	26	31	296	248	41	51
*V0 domain*								
	gi|328777195	p. v-type proton ATPase 116 kDa subunit a isoform 1-like	12	4	117	105	8	16
	gi|328785772	p. v-type proton ATPase 116 kDa subunit a isoform 1-like isoform 1	0	0	16	18	0	4
	gi|328776893	p. probable V-type proton ATPase 116 kDa subunit a-like	6	8	42	27	5	4
	gi|66548758	p. v-type proton ATPase subunit d	14	15	101	124	4	16
	gi|66524947	p. v-type proton ATPase subunit e 2-like	0	2	13	9	0	0
	gi|58585082	vacuolar H+ ATP synthase 16 kDa proteolipid subunit	1	4	27	36	1	4
	gi|48099854	p. v-type proton ATPase 21 kDa proteolipid subunit-like	1	0	11	16	0	0

Spectral counts indicate the peptide numbers of the proteins identified in each gland. The shaded numbers in ‘Spectral count’ column satisfy our frequently detected protein criteria in (n+f) merged counts. p., predicted.

**Fig 4:**
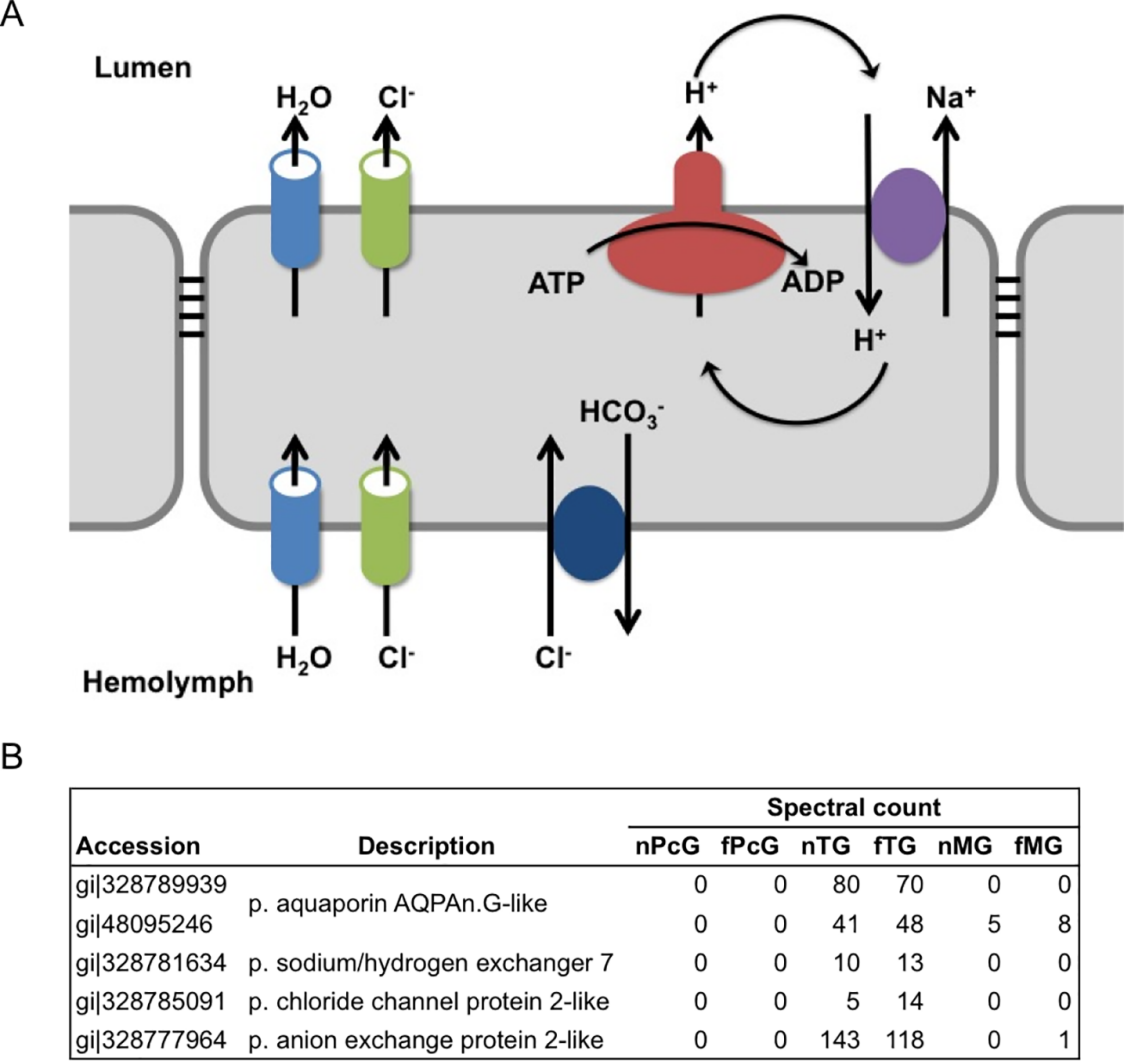
Schematic cartoon and candidate transporters of TG secretory epithelia. (A) The assumed ion transport mechanisms in the insect salivary gland. V-ATPase on the apical membrane is coupled with a cation/proton exchanger on the same side and related to an anion exchanger, aquaporin, and chloride channel. (B) The spectral counts of the related proteins identified from each gland. These transporters satisfy our TG-selective protein criteria. p., predicted.

In the blowfly salivary gland, V-ATPase activity is needed for the secretion of KCl-rich saliva, which is functionally coupled with an unidentified cation/proton antiporter [27]. In addition, in the yellow fever mosquito Malpighian tubules, the transepithelial secretion of NaCl, KCl, and water is driven by V-ATPase, which is also functionally coupled with an unidentified cation/proton exchanger and related to the chloride channel [28]. Yet, these specific transporter proteins have not been identified. To identify these transporters in honeybee TGs, we checked our ‘TG-selective protein’ list and found that aquaporin AQPAn, G-like, sodium/hydrogen exchanger 7, chloride channel protein 2-like, and anion exchange protein 2-like are possible transporters functionally coupled with and/or related to V-ATPases in the TGs (Fig 4A and 4B and S11 Table).

### Functional characterization of the three exocrine glands based on the ‘labor-selective’ proteins

**Fig 5:**
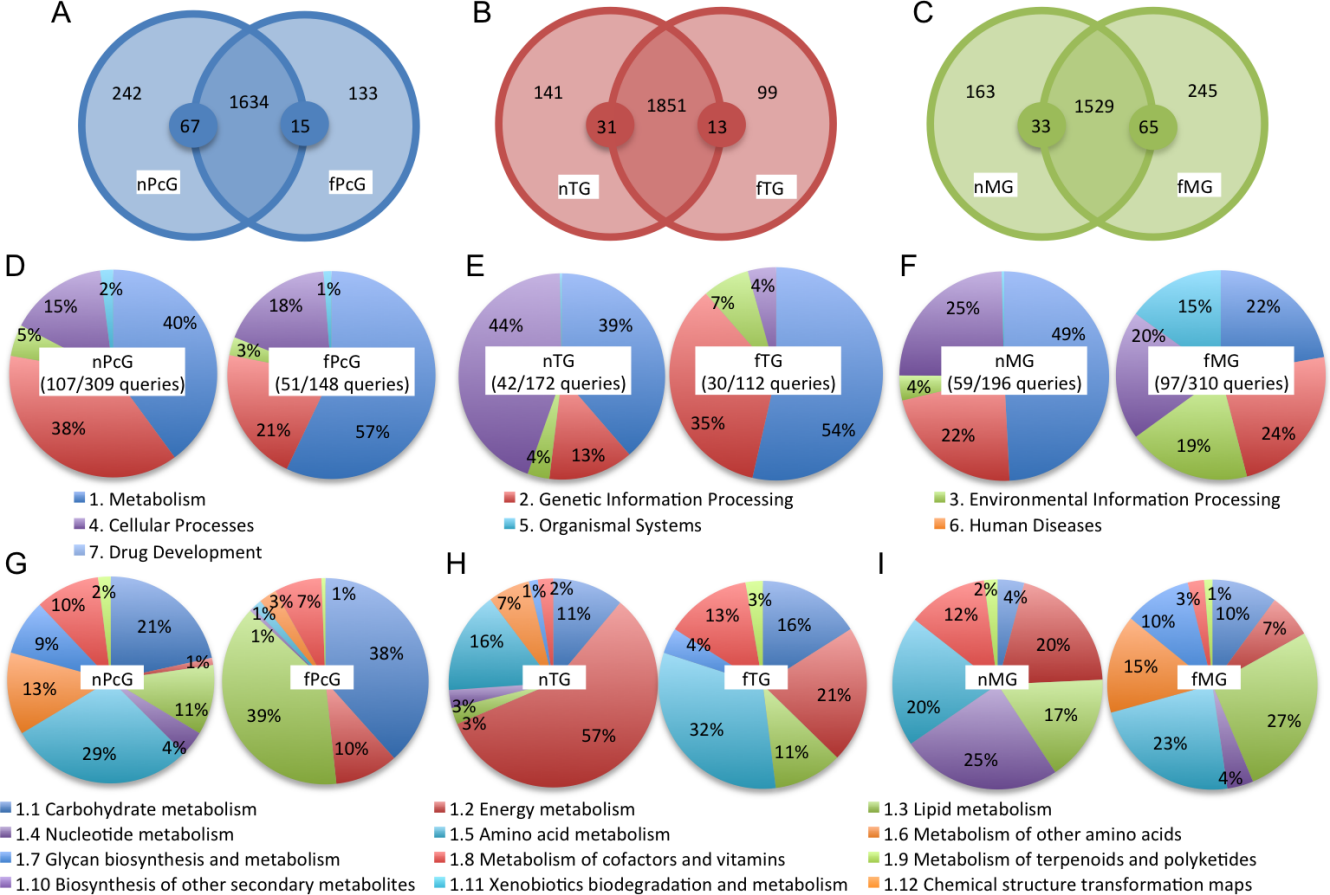
Labor-related functional analysis of the three exocrine glands based on the labor-selective proteins identified from the nPcGs, fPcGs, nTGs, fTGs, nMGs, and fMGs. (A-C) The venn diagrams show the numbers of the proteins specifically detected from the glands of either nurse bees or foragers, and the small circles expanded on the boundaries indicate an abundance at least 5-fold greater than the same-color circles. The number of the corresponding proteins is indicated in each partition. (D-F) Semiquantitative comparison of the KEGG pathway categories of the labor-selective proteins identified from the PcGs (D), TGs (E), and MGs (F). (G-I) Semiquantitative comparison of the KEGG pathway subcategories of the labor-selective proteins categorized as “Metabolism” in panels (D-F).

Next, to understand the ‘labor-selective’ functions of each gland, we focused on ‘labor-selective’ gland proteins by subtracting overlapping proteins identified from those identified from each of the exocrine glands in nurse bees and foragers, and detected 242, 133, 141, 99, 163, and 245 proteins from the nPcGs, fPcGs, nTGs, fTGs, nMGs, and fMGs, respectively (spectral counts>2). The spectral counts of each protein were normalized by the total spectral counts of the gland, and proteins whose spectral counts were more than 5-fold higher than those of the same proteins in the other glands were identified as ‘labor-selective’ proteins of the gland. In total, 309, 148, 172, 112, 196, and 310 ‘labor-selective’ proteins were detected from the nPcGs, fPcGs, nTGs, fTGs, nMGs, and fMGs, respectively (Fig 5A–5C, S14 Table). Next, we performed a pathway analysis using the KEGG Mapper tool with NCBI-gi numbers as queries and mapped 107 among 309 ‘nPcG-selective’, 51 among 148 ‘fPcG-selective’, 42 among 172 ‘nTG-selective’, 30 among 112 ‘fTG-selective’, 59 among 196 ‘nMG-selective’, and 97 among 310 ‘fMG-selective’ proteins, then each enrichment p-value was calculated (S15 Table and S3 Fig).

In both nPcGs and fPcGs, the largest and second largest categories corresponded to “Metabolism” (40% and 57% for nPcG and fPcG) and “Genetic information processing” (38% and 21% for nPcGs and fPcGs), respectively (Fig 5D). In “Metabolism”, the largest subcategories corresponded to “Amino acid metabolism” for nPcGs (29%) and “Lipid metabolism” for fPcGs (39%), suggesting that the corresponding metabolism is upregulated in nPcGs and fPcGs (Fig 5G). The second largest categories of “Metabolism” corresponded to “Carbohydrate metabolism” for both nPcGs and fPcGs (Fig 5G). Notably, the “Amino acid metabolism” subcategory was very small (1%) in fPcGs (Fig 5G), suggesting that drastic changes in metabolism occur in PcGs according to the division of labor of the workers.

In nTGs, “Cellular processes” (44%) and “Metabolism” (39%) corresponded to the largest and second largest categories, whereas, in fTGs, “Metabolism” (54%) and “Genetic information processing” (35%) corresponded to the largest and second largest categories (Fig 5E). As V-ATPase proteins were mapped in both “Metabolism” and “Cellular processes” (S12 Table), we selected V-ATPase proteins for further analysis. We found that whereas most of the V-ATPase proteins were detected in both nTGs and fTGs (Table 3), v-type proton ATPase subunit H isoform 1 was identified as an ‘nTG-selective’ protein (Table 3 and S14 Table), suggesting it has an nTG-selective function.

In nMGs, the largest category corresponded to “Metabolism” (49%), whereas in fMGs, the largest and second largest categories corresponded to “Genetic information processing” (24%) and “Metabolism” (22%) (Fig 5F). In “Metabolism”, the “Energy metabolism” subcategory was larger in nMGs (20%) than in fMGs (7%) (Fig 5I). As in TGs, v-type proton ATPase subunit H isoform 1 was detected as an ‘nMG-selective’ protein (Table 3), suggesting nurse bee-selective function of v-type proton ATPase subunit H isoform 1 in these exocrine glands.

To further characterize the ‘labor-related’ MG functions, we manually checked the ‘labor-selective’ protein list. We found that acyl-CoA synthetase family member 2, mitochondrial-like, partial and acyl-CoA synthetase family member 2, mitochondrial precursor, which are the first-step enzymes of peroxisomal β-oxidation, were more frequently detected in nMGs than in fMGs (S14 Table), coinciding with the fact that nurse bees biosynthesize MG acids, which are constituents of RJ, *de novo* [29]. In addition, we detected odorant binding protein (OBP)-14 precursor as an ‘nMG-selective’ protein (S14 Table), consistent with previous OBP studies [30, 31].

### Cytochrome P450 enzymes as ‘labor-dependent’ and/or ‘gland -selective’ MG proteins

Previous studies demonstrated that many cytochrome P450 (CYP) enzymes are involved in hormone and pheromone biosynthesis in insects [32–34]. The hydroxylation of stearic acid by CYPs differs between worker MGs (ω hydroxylation) and queen MGs (ω-1 hydroxylation) [29]. Therefore, to identify CYPs involved in ‘labor-selective’ and/or ‘gland-selective’ pheromone biosynthesis, we manually checked our protein identification lists, focusing on CYPs. Among the 46 CYPs encoded by the honeybee genome [35–37], we identified 14 in the present study; i.e., CYP6AS3, CYP6AS4, CYP6AS5, CYP6AS7, CYP6AS8, CYP6AS11, CYP6BD1, CYP9Q1, CYP9Q2, CYP9Q3, CYP9S1, CYP336A1, CYP305D1, and CYP315A1 (Table 4). Among them, CYP6SA3 and CYP6AS4 were identified as ‘PcG-selective’ proteins. Both of these CYPs function to metabolize quercetin, a phytochemical constituent in honey [38], suggesting a role for the PcGs in the phytochemical metabolism of nectar.

**Table 4:** Summary of CYPs identified from each gland.

			Spectral count							
BeeBase identifier	Gene name	Accession	nPcG	fPcG	nTG	fTG	nMG	fMG	MG selectivity	Abundance
GB19967	CYP9Q3	gi|328781973	0	0	0	0	0	2	S	-
		gi|328781975								
		gi|328781977								
		gi|48098075								
GB19820	CYP9Q1	gi|48098085	0	0	0	0	0	8	S	-
GB13748	CYP9S1	gi|48098081	4	0	20	19	4	20	-	-
GB11943	CYP305D1	gi|66512130	0	0	0	0	117	40	S	A
GB11754	CYP6AS8	gi|328785290	0	0	0	0	245	249	S	A
		gi|66523006								
GB19797	CYP336A1	gi|328789667	0	0	8	4	0	1	-	-
GB19306	CYP6BD1	gi|328784477	51	61	0	0	0	17	-	-
GB16447	CYP315A1	gi|328784025	0	0	0	0	0	2	S	-
		gi|328784023								
GB15793	CYP6AS4	gi|328785304	22	28	0	0	0	1	-	-
GB11027	CYP6AS11	gi|110762372	0	0	0	0	100	43	S	A
GB15681	CYP6AS3	gi|110765954	14	13	0	0	0	0	-	-
		gi|66565910								
GB17434	CYP6AS5	gi|94158657	0	0	0	0	0	7	S	-
GB18052	CYP6AS7	gi|66522973	8	1	12	4	1	8	-	-
GB17793	CYP9Q2	gi|48098073	0	0	0	0	0	3	S	-

S in the ‘MG selectivity’ column and A in the ‘Abundance’ column satisfy our MG-selective and frequently detected protein criteria, respectively.

In addition, eight CYPs: CYP6AS5, CYP6AS8, CYP6AS11, CYP9Q1, CYP9Q2, CYP9Q3, CYP305D1, and CYP315A1, were detected as ‘MG-selective’ proteins (Table 4). Among them, CYP9Q1, CYP9Q2, and CYP9Q3 are involved in metabolism to detoxify in-hive acaricides and quercetin [39], suggesting a role for the MGs in xenobiotic detoxification. CYP315A1 is a well-studied ecdysteroid biosynthesis enzyme [34], but the role of this enzyme in the MGs is obscure. The functions of CYP6AS8, CYP6AS11, and CYP305D1 have not been characterized, except CYP305D1 expression is induced by acaricides [40]. ‘MG-selective’ CYPs could be related to worker type ω hydroxylation based on their expression profiles. Recently, Malka, *et al*. conducted genome-wide expression analyses in MGs of different castes and social contexts using microarrays and revealed that 17 CYP genes are differentially expressed according to the caste: *Cyp9q1*, *Cyp9q3*, *Cyp6aq1*, *Cyp9s1*, *Cyp305d1*, and *Cyp6as8* are upregulated in workers, whereas *Cyp4a53*, *Cyp336a1*, *Cyp6bd1*, *Cyp6as2*, *Cyp6as13*, *Cyp315a1*, *Cyp6as4*, *Cyp15a1*, *Cyp6as11*, *Cyp4az1*, and *Cyp6bc1* are upregulated in queens, respectively [41]. Among these differentially expressed CYPs, 10 CYPs overlapped with our identified CYPs and included 5 CYPs that were upregulated in workers; CYP6AS8, CYP9Q1, CYP9Q3, CYP9S1, and CYP305D1 (Table 4). We also detected seven CYPs: CYP9Q1, CYP9Q2, CYP9Q3, CYP6AS5, CYP6AS7, CYP6BD1, and CYP315A1 as ‘fMG-selective’ proteins; CYP6AS8 as a frequently detected protein in the MGs; and CYP305D1 and CYP6AS11 as frequently detected proteins in the MGs and a greater than 2-fold frequently detected in nMGs compared with fMGs (Table 4). These findings imply that the MGs have a role in pheromone and hormone biosynthesis as well as in xenobiotic detoxification.

### Validation of the variability of protein expression levels using immunoblotting analyses

**Fig 6:**
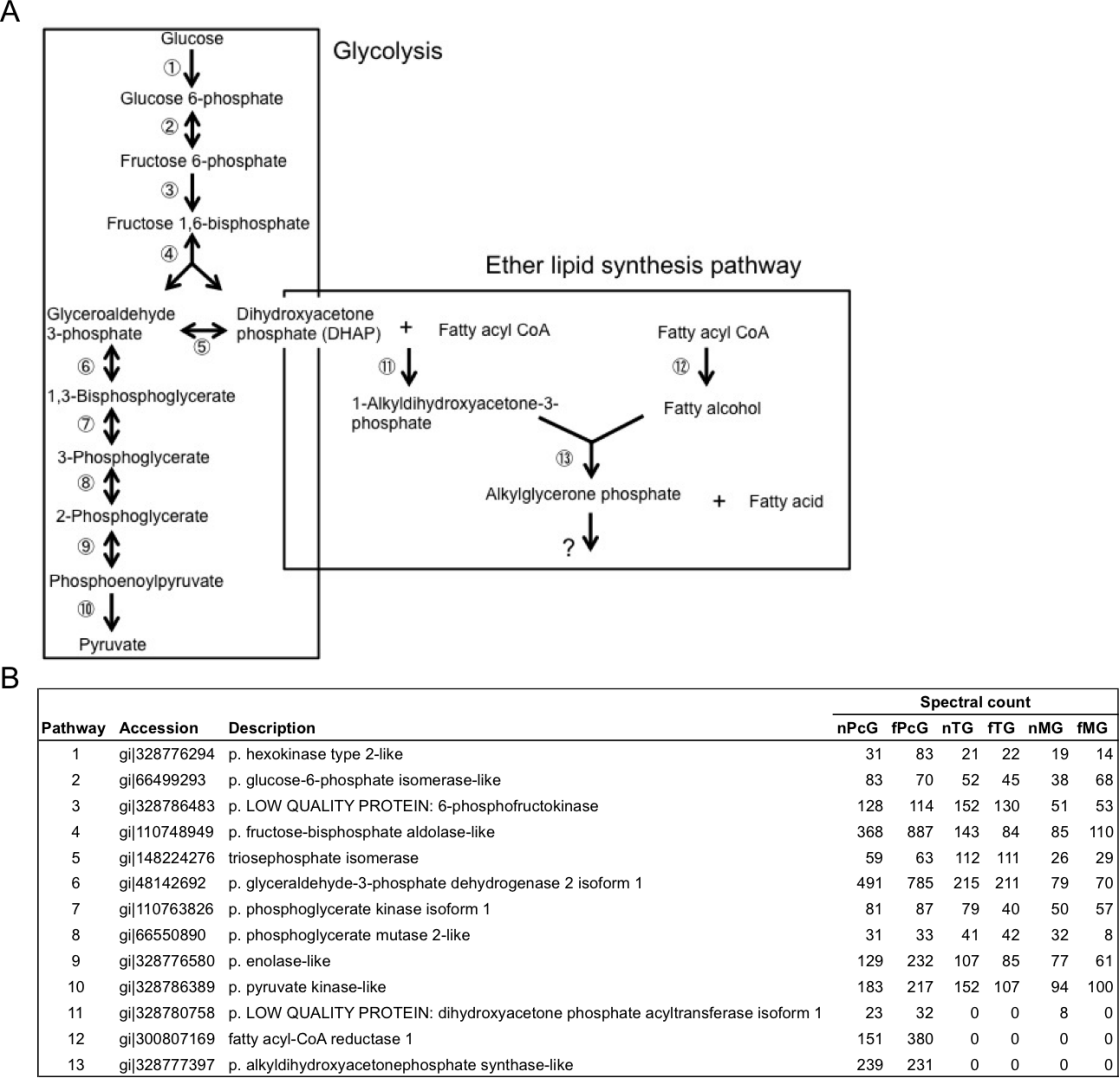
Glycolysis and ether lipid synthesis pathway. (A) The steps of glycolysis and ether lipid synthesis. The numbers indicate enzymes linked to panel (B). The question mark indicates that no homolog is identified in the previous study [49]. (B) The spectral counts of the related proteins identified from each gland. The pathway numbers are linked to panel (A). p., predicted.

Finally, to assess the variability of protein expression levels in the samples analyzed in our proteomic study, we performed immunoblotting analysis using antibodies against four proteins, aldolase, acetyl-CoA acyltransferase 2 (ACAA2), major royal jelly protein (MRJP) 2, and imaginal disc growth factor (IDGF) 4, which we analyzed previously [11], in two independent samples that were newly prepared for this assessment. We selected these four proteins for immunoblotting analyses, because antibodies for aldolase and ACAA2 are commercially available and we previously raised antisera against MRJP2 [13] and IDGF4 [11] in our previous studies [11, 13]. Moreover, these four proteins included both ‘frequently detected’ proteins; aldolase and ACAA2 (Tables 1 and 2, and Fig 6) and ‘not frequently detected’ proteins; MRJP2 and IDGF4, and thus were convenient for validating wide range protein expression level. The spectral counts of most housekeeping proteins, including enzymes for glycolysis (Fig 6A and 6B), however, varied to some extent among the six glands (S7 Table). Therefore, we selected beta-actin, another cytoplasmic housekeeping protein as an internal control protein for immunoblotting. We first performed immunoblotting analyses using antibodies against the above four proteins as well as anti-beta-actin antibodies, and then normalized the band intensities of each protein to that of beta-actin in each gland for each of the two samples. We also normalized the spectral count detected for each of the four proteins by that of beta-actin, and then compared the normalized spectral count for each of the four proteins in each of the six glands with those of the normalized band intensities in the immunoblotting analysis for each of the four proteins in each gland.

**Fig 7:**
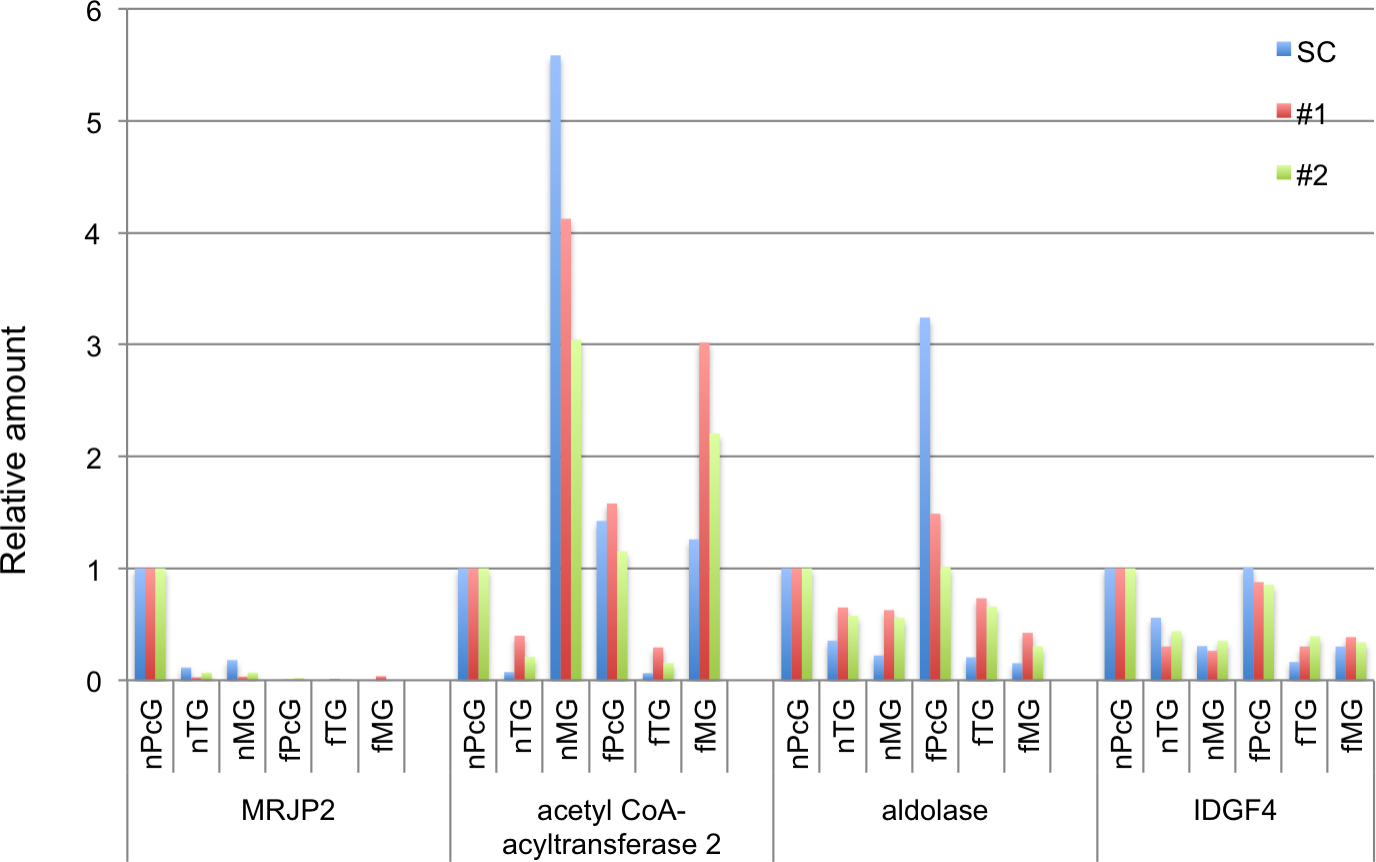
Validation of variable protein expression levels based on immunoblotting analyses. Immunoblotting analysis for MRJP2, aldolase, acetyl-CoA acyltransferase 2, and IDGF4 were performed with two independent biologic samples (red and green bars), and the band intensity for each protein in each gland was normalized to that of beta-actin. The normalized protein expression levels in each gland were compared with the spectral counts (SC) of each protein in each gland normalized to that of beta-actin (blue bars), taking the protein expression levels and spectral counts of nPcG as 1.

The results first indicated that our immunoblotting analysis data are of relatively high reproducibility (S4 Fig), and the variability of the normalized band intensities for each of the four proteins in each gland was at most three-fold; e.g., nTG and fTG for acetyl CoA-acyltarnsferase 2, and nMG and fMG for aldolase, between the two biologic samples (Fig 7). In addition, the normalized band intensities for each of the four proteins in each gland for the two biologic samples were almost comparable to the normalized spectral counts for each protein in each gland (Fig 7), although the variation among the samples was greater in the normalized spectral counts than in the normalized band intensities of the immunoblotting analysis, probably reflecting the less linearity inherent in immunoblotting analysis. These results suggest that the variation of protein expression levels in our biologic samples (replicates) could be less than three-fold, which supports our research strategy focusing on differential protein expression levels of at least five-fold in our proteomic analysis.

## Discussion

### ‘Gland-selective’ functions of worker PcGs

Simpson (1960) previously observed that the secretion of the PcGs is oily and the pH of the PcG lysate is 6.5–7.0 [42]. Morphologic and chemical composition studies revealed that the secretion of neutral lipids from PcGs increases with the division of labor of workers; the highest amount of secretion and the largest alveolar size are detected in forager PcGs [43–47], suggesting that forager PcGs secrete neutral lipids.

In honeybees, EO is a primer pheromone that is synthesized *de novo* by foragers from glucose to affect the onset of nurse bee’s foraging, and is detected in the labial gland (another term used for the PcGs) [21]. We previously used shotgun proteomics to show that the PcG proteome reflects its function as an EO synthetic organ [11, 12]. Our previous [11, 12] and present findings that both aldolase, which is a glycolysis enzyme that catalyzes fructose 1,6-bisphosphate cleavage to produce DHAP, and alkyl-DHAP synthase-like, which catalyzes the second step of the ether lipid pathway to exchange the fatty acid added to DHAP with a long-chain fatty alcohol, then free fatty acid is synthesized [48], are frequently detected and prominent in PcGs strongly suggest that the “Ether lipid metabolism” pathway, which is connected with glycolysis is upregulated in PcGs (Fig 6A and 6B). Recently, Faust et al. [49] provided a peroxisomal-related protein inventory in fruit flies, including ether lipid pathway enzymes. We compared the inventory and our KEGG-mapped list, and found that both dihydroxyacetone phosphate acyltransferase isoform 1 (DHAP acyltransferase) and fatty acyl-CoA reductase 1 (FAR) are also ‘PcG-selective’ and 1.3-fold (DHAP-acyltransferase) and 2.5-fold (FAR) more frequently detected in fPcGs than in nPcGs (Fig 6A and 6B), further supporting our notion that the “Ether lipid metabolism” function is upregulated and oleic acid production in fPcGs.

### ‘Gland-selective’ functions of worker TGs

Simpson (1960) also observed that the TGs secrete aqueous saliva, and the pH of the TG lysate is 6.0–6.5 [42]. In addition, studies of TG morphology and labor-related morphologic features revealed that TG secretory tubes are composed of two types of tube cells: secretory and parietal tube cells, the latter of which do not show morphologic changes related to the division of labor of workers [50, 51]. TG secretory tube cells have a well-developed basal labyrinth, abundant rough endoplasmic reticulum, and poor smooth endoplasmic reticulum, suggesting that TGs have a high rate of fluid transport through the cells and produce no pheromones, and only some proteins [50].

In the TGs, “Energy metabolism” was the largest subcategory of “Metabolism” (Figs 2B and 3B), which is again consistent with our previous study using shotgun proteomics [12]. In the”Energy metabolism” category, almost all V-ATPases were ‘TG-selective’ proteins (Table 3 and S11 Table). V-ATPase is a large multi-subunit complex ATP-dependent proton pumps composed of an ATP-hydrolytic domain (V_1_) and a proton-translocation domain (V_0_) [25]. Every eukaryotic cell has V-ATPases in the intracellular acid organelle membranes and/or the plasma membrane, which regulate the pH of intracellular organelles and diverse cellular activities; e.g., acidification of the extracellular space, secretion or reabsorption of ions and fluids, and importing of nutrients [25].

Studies using the blowfly *Calliphora vicina* salivary gland, the yellow fever mosquito *Aedes aegypti* Malpighian tubules, and the tobacco hornworm *Manduca sexta* larval midgut as model systems revealed that V-ATPase resides mainly in the apical plasma membrane in insect epithelia [27, 28, 52, 53]. In these epithelia, cation transport is driven by the V-ATPase cation/H^+^ exchanger and other functionally coupled and/or related transporters.

In the present study, based on our ‘TG-selective protein’ list, we propose aquaporin AQPAn, G-like, sodium/hydrogen exchanger 7, chloride channel protein 2-like, and anion exchange protein 2-like as possible transporters functionally coupled with and/or related to V-ATPases in the TGs (Fig 4A and 4B). Further biochemical analyses are needed to clarify the kinds of solutes transported in TG epithelia.

### ‘Gland-selective’ functions of worker MGs

The role of honeybee MGs in pheromone production has been extensively studied [2]. Queen MGs produces 9-keto-(E)-2-decenoic acid and 9-hydroxyl-(E)-2-decenoic acid, both of which are major components of the queen pheromones that suppress the development of worker ovaries [2, 54–58], whereas worker MGs produce 2-heptanone as a major component of the alarm pheromone [59] and 10-hydroxyl-(E)-2-decenoic acid as a major lipid component of RJ [60]. Hasegawa *et al*. previously demonstrated that fatty acid synthase (FAS) is selectively expressed in worker MGs but not in queen MGs, and proposed that worker-selective FAS expression is responsible for the differential metabolism of fatty acids between workers’ and queen MGs [61]. Actually, in the present study, FAS was identified as the most frequently detected protein in the MGs and the third most frequently detected protein in the PcGs (Table 1), further supporting the notion that FAS functions in pheromone biosynthesis in the MGs.

Among 14 CYPs detected in the present study, 2 CYPs were ‘PcG-selective’ proteins, whereas 8 CYPs were ‘MG-selective’ proteins (Table 4 and S11 Table). It might be that, while ‘PcG-selective’ CYPs are involved in the phytochemical metabolism of nectar, ‘MG-selective’ CYPs are related to worker type ω hydroxylation, considering their expression profiles. Recently, Malka, *et al*. conducted genome-wide expression analyses of MGs among different castes and social contexts using microarrays, and revealed that 17 CYPs are differentially expressed according to the caste [41]. Among these differentially expressed CYPs, 10 CYPs overlapped with our identified CYPs and included 5 CYPs that were upregulated in workers; CYP6AS8, CYP9Q1, CYP9Q3, CYP9S1, and CYP305D1 (Table 4). These frequently detected CYPs that were upregulated in workers, especially CYP305D1, which was worker upregulated [41], MG-selective, frequently detected, and with greater than 2-fold higher abundance in the nMGs than fMGs (Table 4 and S14 Table), are promising candidates for the worker type ω hydroxylation enzyme. Recently, Wu et al. used RNA sequencing and quantitative reverse-transcription polymerase chain reaction analyses to suggest that *CYP6AS8* and *CYP6AS11* are responsible for the different hydroxylation processes in worker and queen MGs, respectively [62], which coincides well with our proteomics data. Further functional studies will be fruitful for elucidating the molecular basis of pheromone biosynthesis in the MG.

### ‘Labor-selective’ functional changes of the three exocrine glands: PcGs, TGs, and MGs

We also analyzed the ‘labor-dependent’ functions of each gland. Our previous [12] and present KEGG pathway analyses suggested that the major function of PcGs changes from amino acid metabolism to lipid metabolism according to the division of labor of workers (Fig 5G). All identified ether lipid synthesis pathway proteins were PcG-selective and some were more frequently detected in fPcGs than in nPcGs, suggesting that the ether lipid synthesis pathway was upregulated in the PcGs, especially the fPcGs. In addition, trehalase precursor and facilitated trehalose transporter Tret 1-like were identified as fPcG-selective proteins (S14 Table), implying that trehalose transport and consumption in fPcGs is upregulated in the hemolymph. These findings strongly supported the notion that fPcGs synthesize EO. In our previous study, MRJP 1, 2, and 4 were identified from PcGs [12]. In the present study, we additionally identified MRJP 3, 5, and 7 as nPcG-selective proteins. These MRJPs in RJ are also synthesized by PcGs (S14 Table).

Although there were only small numbers of proteins detected as ‘labor-selective’ proteins in the TGs (S14 Table), v-type proton ATPase subunit H isoform 1 was identified as an ‘nTG-selective’ protein among various V-ATPase proteins (Table 3 and S14 Table), suggesting that different ATPase subunit isoforms comprise the ATPase complexes in nTGs and fTGs. V-ATPase subunit H is a regulatory subunit of the V-ATPase complex and overexpression of the gene for V-ATPase subunit H affects life span in the fruit fly [63]. In the Oriental migratory locust (*Locusta migratoria manilensis*), *Vhasfd*, a gene for V-ATPase subunit H is essential for survival and molting [64]. Likewise, transketolase isoform 2 was identified as an ‘fTG-selective’ protein, although transketolase isoform 1 was not detected as a ‘labor-selective’ protein (S14 Table). Although the biologic significance of the differential expression of the v-type proton ATPase subunit H isoform 1 and transketolase isoform 2 between nTGs and fTGs is presently obscure, these might be related to the differential TG secretion capacity between nurse bees and foragers.

Among the three exocrine glands, the largest number and ratio of ‘labor-selective’ proteins and spectral counts were identified in the MGs (Fig 5C and S14 Table). Our findings suggest selective upregulation of β-oxidation, which might be related to RJ lipid synthesis, in the nMGs. In addition, we detected OBP 14 precursor as an ‘nMG-selective’ protein (S14 Table). Previous studies demonstrated that, while OBP 14 is abundantly expressed in worker larvae and in-hive worker MGs, it is expressed at lower levels in newly emerged and forager bee MGs [30, 31], consistent with the present results. Insect OBPs are soluble proteins that function in both recognizing chemical stimuli at the sensory nerve and in releasing chemicals from the exocrine glands [65]. In addition, we and other groups also detected OBP 14 in RJ [12, 66]. These findings suggest that nMGs secrete OBP 14 as a protein component of RJ and/or an RJ ingredient-releasing carrier. Future biochemical and functional analyses of ‘gland-selective’ and ‘labor-selective’ proteins are needed to clarify the biologic relevance of these exocrine gland proteins in honeybees.

## Materials and methods

### Animals and dissection of organs

European honeybees (*A*. *mellifera* L.) were purchased from a local dealer (Kumagaya Youhou, Saitama, Japan) and maintained at the University of Tokyo (Hongo Campus, Bunkyo-ku, Tokyo). Nurse bees and foragers were collected separately based on their behaviors and the development of the HpGs, as described previously [12]. Briefly, in-hive workers with well-developed HpGs that repeatedly inserted their heads into honeycombs containing larvae were collected as nurse bees, and those with shrunken HpGs and pollen loads on their hind legs were collected as forager bees. The MGs, PcGs, and TGs were dissected under a stereomicroscope from the heads and thoraxes of bees each after first anesthetizing the workers on ice. For mass spectrometric analysis, glands from 10 nurse bees and 8 foragers were pooled and analyzed. For the immunoblotting analysis, glands from 10 nurse bees and 10 foragers were pooled and analyzed. Dissected glands were frozen and stored at -80ºC until use.

### Protein extraction

Frozen samples were homogenized manually with disposable pestles in plastic tubes containing lysis solution: 8 M urea, 2% 3-[(3-cholamidopropyl)-dimethylammonio]-1-propanesulfonate, 0.28% dithiothreitol, and 0.5% IPG buffer (pH 3–10, GE Healthcare Bioscience). After the lysate was centrifuged, the supernatants were collected and proteins were precipitated with 10% trichloroacetic acid. The precipitants were dissolved in rehydration solution: 2M Tris-HCl, pH 8.2, containing 8 M urea. The protein amounts were determined using a BCA Protein Assay Kit (Pierce Chemical).

### Mass spectrometric analysis

The protein extracts (10 μg/sample) were subjected to shotgun proteomics using a direct nanoflow liquid chromatography-tandem mass spectrometry (LC-MS/MS) system, as described previously [67]. Briefly, the extracts were digested with 25 pmol of trypsin, desalted using ZipTip C_18_ (Millipore, Billerica, MA), concentrated, and injected into a direct nanoflow liquid chromatography system (DiNa-2A, KYA Technologies, Tokyo, Japan) coupled to the LTQ-Orbitrap Velos mass spectrometer (Thermo Fisher Scientific, Bremen, Germany), as described previously [67]. After applying the peptide mixture to a C_18_ column (800 μm inner diameter x 3 mm long), reversed-phase separation of the captured peptides was performed using a column (150 μm inner diameter x 150 mm long) filled with HiQ sil C_18_ (3 μm particles, 120Å pore; KYA Technologies, Tokyo, Japan). The peptides were eluted with a linear 2–40% gradient of acetonitrile containing 0.1% formic acid at a flow rate of 300 nl/min. Mass spectra were acquired in data dependent mode, switching automatically MS and MS/MS acquisition. All full-scan MS spectra in the range from *m*/*z* 380 to 2000 were acquired in the FT-MS part of the mass spectrometer with a target value of 1,000,000 and a resolution of 100,000 at *m*/*z* 400. The 20 most intense ions that satisfied an ion selection threshold above 2000 were fragmented in the linear ion trap with normalized collision energy of 35% for activation time of 10 ms. For accurate mass measurement, the Orbitrap analyzer was operated with the “lock mass” option using polydimethylcyclosiloxane (*m*/*z* = 445.120025) and bis(2-ethylhexyl) phthalate ions (*m*/*z* = 391.284286).

### Protein identification

The acquired MS/MS signals were processed with Proteome Discoverer (version 1.3, Thermo Fisher Scientific) and searched against a dataset of honeybee RefSeqs using the Mascot algorithm (version 2.4.1, Matrix Science) with the following parameters; fixed modifications: carbamidomethylation (cysteine); variable modifications: oxidation (methionine), acetylation (protein N-term), and pyro-glutamination (N-terminal glutamine); maximum missed cleavages: 2; peptide mass tolerance: 3 ppm; and MS/MS tolerance: 0.8 Da; as described by Narushima *et al*. [67]. For peptide identification, we conducted decoy database searches using Mascot and applied a filter to satisfy a false positive rate of less than 1% [68]. The protein sequences determined by more than two MS/MS spectra in the whole measurement were then referred to as identified proteins. The mass spectrometry proteomics data have been deposited to the ProteomeXchange Consortium via the jPOST repository with the dataset identifier PXD005918 [69, 70].

### Data analysis

The identified protein lists were attached with the NCBI-gi accession numbers and corresponding amino acid sequences. The pathway analysis was performed as described previously [12]. Briefly, using the Kyoto Encyclopedia of Genes and Genomes (KEGG) Mapper tool (http://www.genome.jp/kegg/tool/map_pathway2.html), NCBI-gi numbers were applied as queries, acquired pathway search results were grouped under the KEGG Pathway Maps. Enrichment p-values were calculated based on hypergeometric distribution. Mapping was performed twice, and the results were merged together. In each category, the spectral counts were summed for the semiquantitative analyses [12].

### Immunoblotting and image quantitation

Immunoblotting analyses were performed essentially as described previously [11]. Two lots of independent samples of PcGs, TGs and MGs, dissected from 10 nurse bees and 10 foragers, were homogenized and lysed in the aforementioned lysis solution, subjected to TCA precipitation, and then re-lysed in SDS-sample buffer (150 mM Tris-HCl, pH 6.8, containing 1.2% SDS and 30% glycerol). Immunoblotting analyses were performed using anti-IDGF4 antiserum [11], anti-human acetyl-CoA acyltransferase 2 rabbit polyclonal antibody (Sigma-Aldrich), anti-human aldolase rabbit polyclonal antibody (Cell Signaling Technology), and anti-50kDa protein (also known as MRJP2) antiserum [13], and anti-beta-actin mouse monoclonal antibody (Santa Cruz Biotechnology) as an internal control, and horseradish peroxidase-conjugated anti-rabbit IgG or anti-mouse IgG as secondary antibodies, respectively. Immunoreactive proteins were detected with a chemiluminescence reagent, ECL Select (GE Healthcare) and images were acquired by ImageQuant LAS 4000mini (GE Healthcare). Signal intensity quantification was performed using ImageJ (https://imagej.nih.gov/ij/).

## Supporting information

S1 Fig Enrichment analysis of frequently detected proteins identified from PcG, TG and MG. Panels A and B represent -log_10_ (enrichment p-value) of each KEGG pathway category (A) and subcategory (B), respectively.
(PDF)

S2 Fig Enrichment analysis of gland-selective proteins identified from PcG, TG and MG. Panels A and B represent -log_10_ (enrichment p-value) of each KEGG pathway category (A) and subcategory (B), respectively.
(PDF)

S3 Fig Enrichment analysis of labor-selective proteins identified from PcG, TG and MG. Panel A and B represent -log_10_ (enrichment p-value) of each KEGG pathway category (A) and subcategory (B), respectively.
(PDF)

S4 Fig Immunoblotting analyses to validate the variability of protein expression levels in each gland. Panel A-D and E-H indicate the results for sample #1 and #2, respectively. Immunoblotting analysis was performed with anti-50kDa protein antiserum (A, E), anti-ACAA2 antibodies (B, F), anti-aldolase antibodies (C, G), and anti-IDGF4 antiserum (D, H). The lower panels indicate the results for immunoblotting analysis using anti-beta-actin antibodies as an internal control for the result indicated in each upper panel. P: PcG, T: TG, and M: MG, N: nurse bee; F: forager. The arrows indicate bands for MRJP2 (A, E), ACAA2 (B, F), aldolase (C, G) and IDGF4 (D, H), respectively. All lanes were loaded with 20 μg protein equivalent.
(PDF)

S1 Table List of the nPcG proteome. (XLSX)

S2 Table List of the fPcG proteome. (XLSX)

S3 Table List of the nTG proteome. (XLSX)

S4 Table List of the fTG proteome. (XLSX)

S5 Table List of the nMG proteome. (XLSX)

S6 Table List of the fMG proteome. (XLSX)

S7 Table Semi-quantitative summary of the total proteins identified from nPcG, fPcG, nTG, fTG, nMG and fMG. (XLSX)

S8 Table List of the frequently detected proteins identified from PcG, TG and MG. (XLSX)

S9 Table List of the KEGG-mapped frequently detected proteins identified from PcG, TG and MG. Enrichment p-values were calculated based on hypergeometric distribution.
(XLSX)

S10 Table List of the frequently detected proteins categorized in ‘Carbohydrate metabolism’ from the three exocrine glands. (XLSX)

S11 Table List of the gland-selective proteins identified from PcG, TG and MG. (XLSX)

S12 Table List of the KEGG-mapped gland-selective proteins identified from PcG, TG and MG. Enrichment p-values were calculated based on hypergeometric distribution.
(XLSX)

S13 Table List of the identified proteins relative to F1F0 ATP synthase from the three exocrine glands. (XLSX)

S14 Table List of the labor-selective proteins identified from nPcG, fPcG, nTG, fTG, nMG and fMG. (XLSX)

S15 Table List of the KEGG-mapped labor-selective proteins identified from nPcG, fPcG, nTG, fTG, nMG and fMG. Enrichment p-values were calculated based on hypergeometric distribution.
(XLSX)
