# Supplementary figures and images for: Shotgun proteomics deciphered age/division of labor-related functional specification of three honeybee (Apis mellifera L.) exocrine glands
Source: PLoS One. 2018 Feb 15;13(2):e0191344. doi: 10.1371/journal.pone.0191344 (PMC5813902; doi:10.1371/journal.pone.0191344)

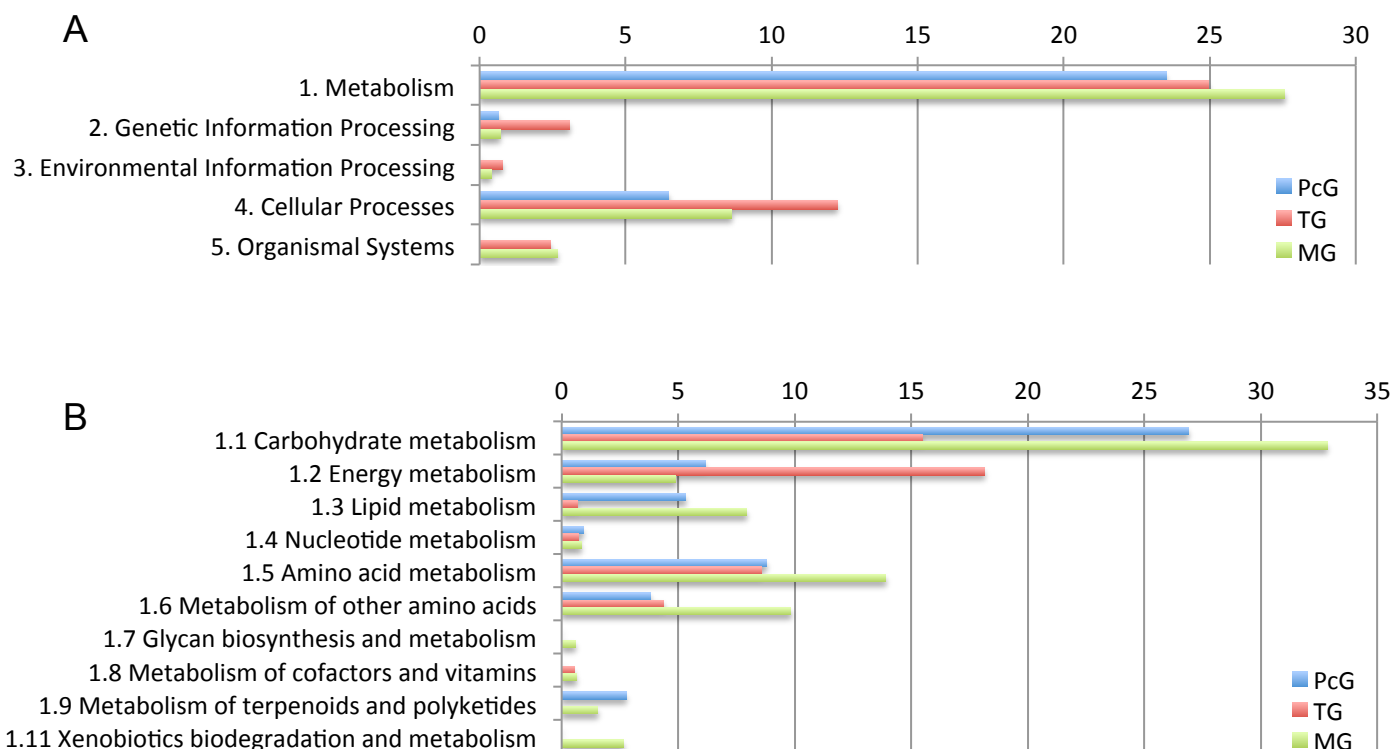

**Figure S1**

Supplement: S1 Fig — Panels A and B represent -log10 (enrichment p-value) of each KEGG pathway category (A) and subcategory (B), respectively. (PDF) [file pone.0191344.s001.pdf]

A

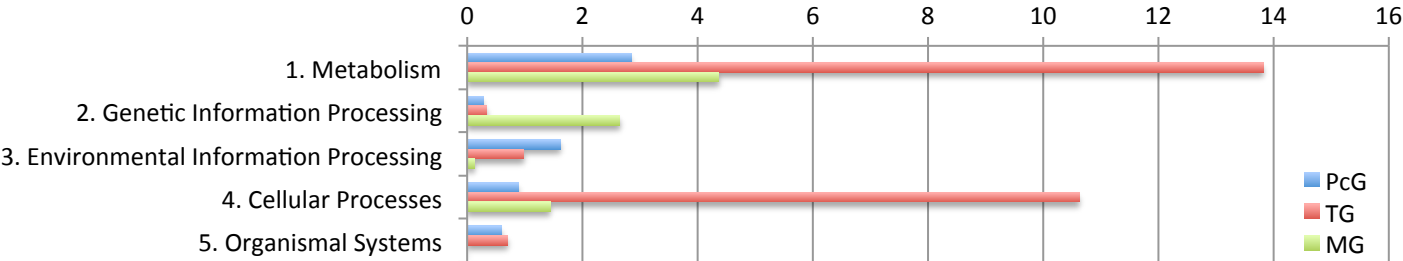

B

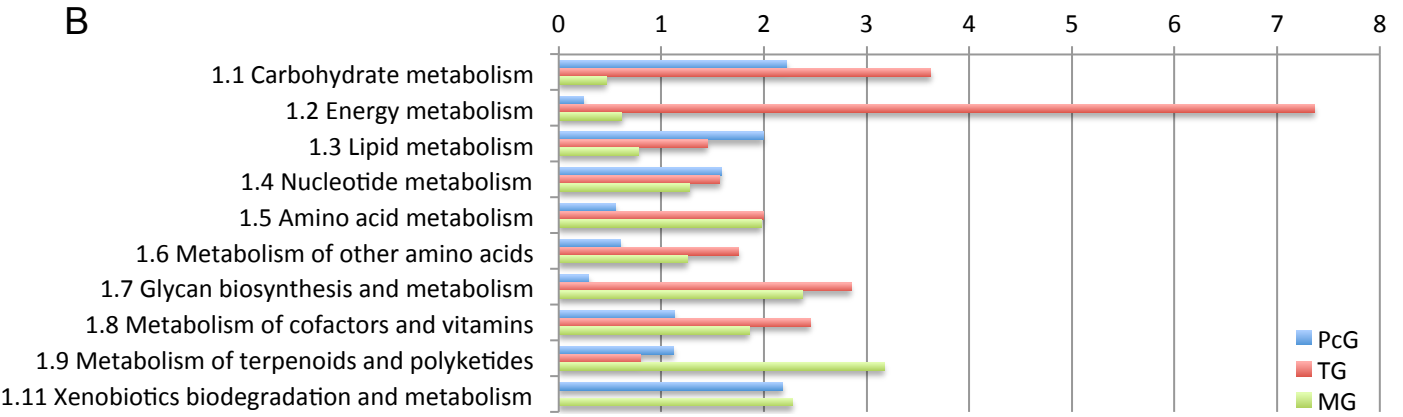

Figure S2

Supplement: S2 Fig — Panels A and B represent -log10 (enrichment p-value) of each KEGG pathway category (A) and subcategory (B), respectively. (PDF) [file pone.0191344.s002.pdf]

A

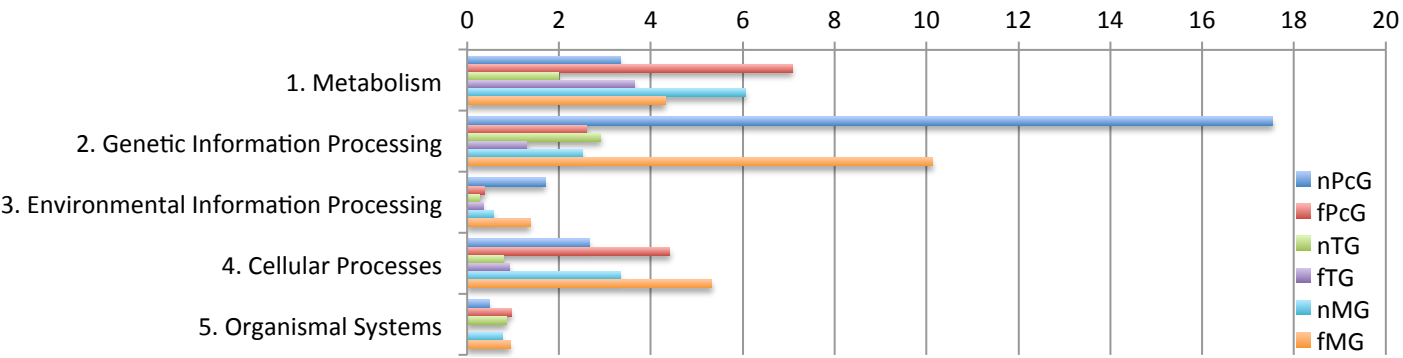

B

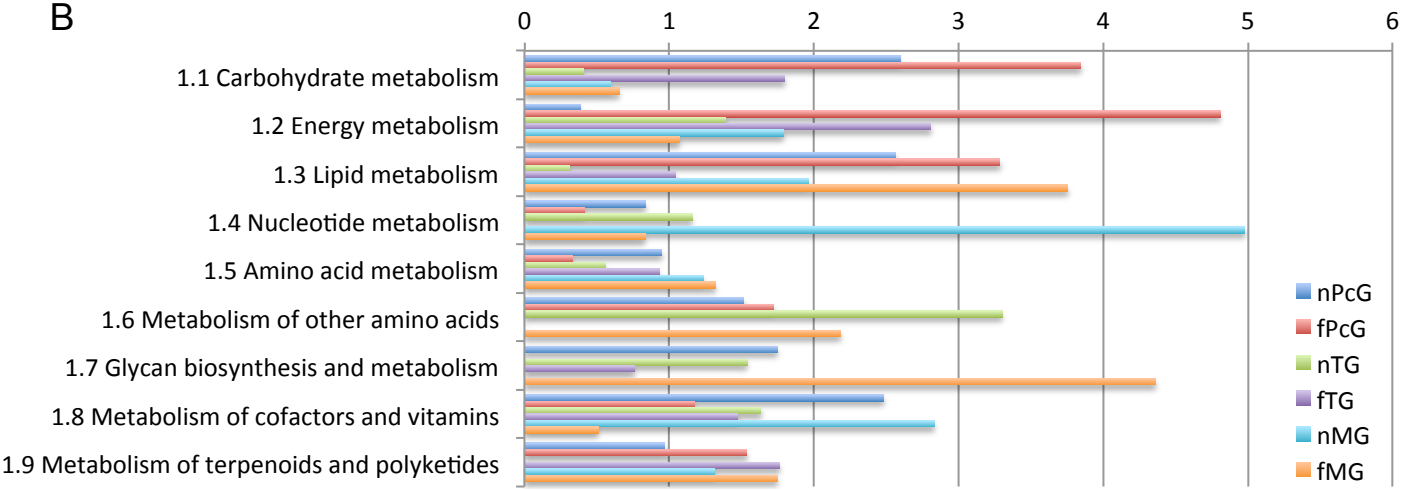

Figure S3

Supplement: S3 Fig — Panel A and B represent -log10 (enrichment p-value) of each KEGG pathway category (A) and subcategory (B), respectively. (PDF) [file pone.0191344.s003.pdf]
