# Supplementary material for: Shotgun proteomics deciphered age/division of labor-related functional specification of three honeybee (Apis mellifera L.) exocrine glands
Source: PLoS One. 2018 Feb 15;13(2):e0191344. doi: 10.1371/journal.pone.0191344 (PMC5813902; doi:10.1371/journal.pone.0191344)
Supplement: S4 Fig — Panel A-D and E-H indicate the results for sample #1 and #2, respectively. Immunoblotting analysis was performed with anti-50kDa protein antiserum (A, E), anti-ACAA2 antibodies (B, F), anti-aldolase antibodies (C, G), and anti-IDGF4 antiserum (D, H). The lower panels indicate the results for immunoblotting analysis using anti-beta-actin antibodies as an internal control for the result indicated in each upper panel. P: PcG, T: TG, and M: MG, N: nurse bee; F: forager. The arrows indicate bands for MRJP2 (A, E), ACAA2 (B, F), aldolase (C, G) and IDGF4 (D, H), respectively. All lanes were loaded with 20 μg protein equivalent. (PDF) [file pone.0191344.s004.pdf]

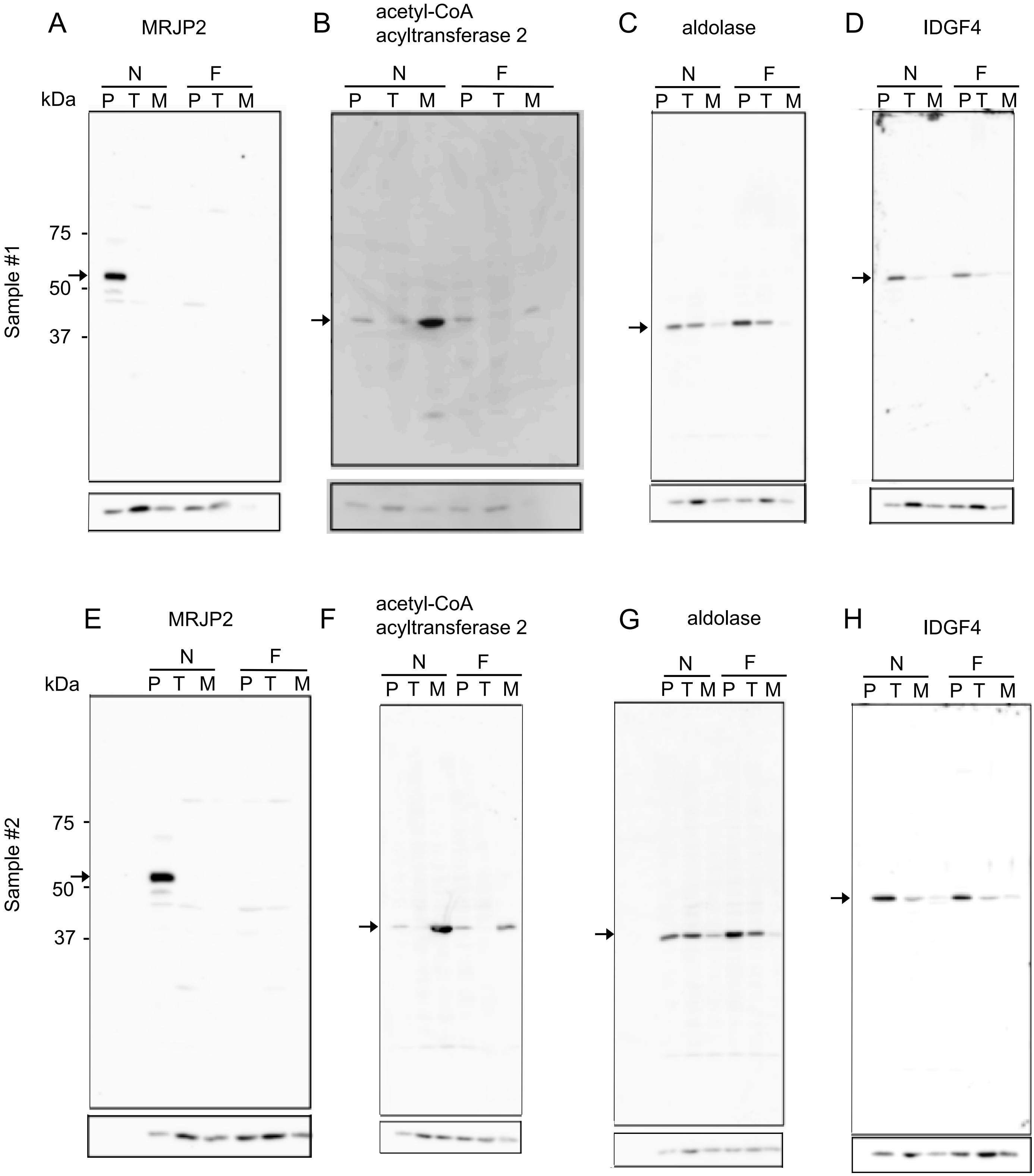
A
MRJP2
B
acetyl-CoA acyltransferase 2
C
aldolase
D
IDGF4
N F
kDa
P T M P T M
75
50
37
Sample #1
E
MRJP2
F
acetyl-CoA acyltransferase 2
G
aldolase
H
IDGF4
Sample #2

Figure S4
